# The efficacy and safety of fast track surgery (FTS) in patients after hip fracture surgery: a meta-analysis

**DOI:** 10.1186/s13018-021-02277-w

**Published:** 2021-02-27

**Authors:** Mingyang Jiang, Siyi Liu, Huachu Deng, Xuzhi Liang, Zhandong Bo

**Affiliations:** 1 Department of Bone and Joint Surgery, The First Affiliated Hospital of Guangxi Medical University, Nanning, Guangxi China; 2 Guangxi Medical University, Nanning, Guangxi China

**Keywords:** Fast track surgery (FTS), Enhanced recovery after surgery (ERAS), Hip replacement, Meta-analysis

## Abstract

**Background:**

Fast track surgery (FTS) has been gradually applied in perioperative management of orthopedic surgery, but there still some research suspected that the prognosis of patients is not as expected and the cost is high, the effect of the FTS still urgently needed for support by evidence-based medicine.

**Methods:**

We retrieved RCTs from medical research literature databases. Risk ratios (RR), standard mean difference (SMD), and 95% confidence intervals (CI) were calculated to compare the primary and safety endpoints.

**Results:**

Overall, a total of 8886 patients were retrieved from 57 articles, of which 4448 patients (50.06%) were randomized to experimental group whereas 4438 patients (49.94%) were randomized to control group. The result showed that FTS could significantly shorten the length of stay (LOS), decrease the visual analog scale (VAS), reduce the leaving bed time and the hospitalization costs, and improve Harris hip joint function score. The incidence of complications such as respiratory system infection, urinary system infection, venous thrombus embolism (VTE), pressure sore, incision infection, constipation, and prosthesis dislocation also has been decreased significantly. Meanwhile, FTS improved patients’ satisfaction apparently.

**Conclusions:**

This meta-analysis reveals that FTS could significantly shorten the length of stay, alleviate the pain, reduce the leaving bed time and the hospitalization costs, and improve hip function. The incidence of complications also has been decreased significantly. Meanwhile, FTS has been spoken highly in patients in terms of nursing satisfaction. Its efficacy and safety were proved to be reliable.

**Supplementary Information:**

The online version contains supplementary material available at 10.1186/s13018-021-02277-w.

## Introduction

Hip fracture is a public health problem that could come with number of complications even threaten your life [1]. It could happen in any age and most common caused by falling [2]. Surgery is the most common treatment, and the rehabilitation therapy is being encouraged in order to avoid complications and resume routine activities of life. With the increase of age, the realization of rehabilitation plan is more limited. Especially with other basic diseases, the difficulty of rehabilitation increases, and the recovery of hip fracture may be limited [3].

Fast track surgery (FTS), also known as enhanced recovery after surgery (ERAS), is a new surgical concept aiming at early ambulation, discharge, and return to activities of daily living [4, 5]. It uses a series of perioperative optimization measures confirmed by evidence-based medicine to eliminate the factors that delay postoperative recovery [6]. There are 3 parts of FTS: preoperative, intraoperative, and postoperative [7]. Psychological comfort and physical muscle training might reduce patients’ response to psychological and surgical stress preoperative; intraoperative, optimizing anesthesia, and procedure (minimally invasive surgery, MIS), which can reduce pain and shorten recovery time; postoperative nutrition support and pain management are beneficial to improve organ dysfunction, and early rehabilitation exercise could prevent surgery-related complications [8–13].

Currently, the concept of FTS has been widely used in surgical malignant tumors and laparoscopic surgery (especially colorectal surgery), which is expected to be extended to other surgical specialties safely and effectively [14]. Though it is also gradually applied to the perioperative management of orthopedic surgery, the effect of perioperative in elderly patients with hip fracture is still controversial [15, 16]. Eriksson et al. pointed out that there was no difference in mortality or length of stay (LOS) between the FTS group and the standard group [17]. The result that the incidence of adverse events (AEs) in FTS group was less than standard group has no statistical significance. According to the research conducted by Hansson et al., the FTS could shorten the time to operation, which is the only significant difference between FTS group and standard group, but there is no difference in the LOS or the incidence of AEs [18]. Haugan et al. also showed that there was no statistically significant difference in mortality and readmission rates between the FTS and the standard care models for first-time admission [19]. All in all, the data of FTS in the rehabilitation and prognosis of elder patients did not meet the expectations.

The purpose of this article is to analyze the effect of nursing intervention based on FTS concept on perioperative pain management, postoperative length of stay and incidence of complications of hip fracture in the elderly by meta-analysis. It could provide effective evidence for perioperative nursing of hip fracture in the elderly.

## Methods

### Search strategy

Published articles were searched by two researchers for comparing the efficacy and safety of fast-track surgery (FTS) in elderly patients with hip fracture following the Preferred Reporting Items for Systematic Reviews and Meta-Analyses (PRISMA) guidelines [20]. We have searched the RCTs systematically in the databases such as the Cochrane Library, Embase, PubMed, Google Scholar, Baidu Scholar, CNKI, and VIP with no restrictions on language or publication date from January 1, 2015, to August 1, 2020. The following keywords and MeSH terms were used: ('enhanced recovery' or 'ERAS' or 'fast track surgery' or 'accelerated rehabilitation' or 'accelerated care') and 'hip fracture' and 'standard care'. Additional relevant studies were retrieved from reviews, meta-analyses, and other literature. Two authors screened and double-reviewed the retrieved studies. In the event of a dispute, it shall be settled by the third author. In this meta-analysis, all data were extracted from previously published studies, thus patient consent and ethical approval were not required.

### Inclusion and exclusion criteria

The following inclusion criteria were used: (1) studies that assessed the efficacy and safety of FTS in elderly patients with hip fracture, (2) the study was a randomized controlled trial (RCT), (3) the study subjects were elderly patients undergoing hip fracture, (4) general information (e.g., gender, age, disease type) of the experimental group and the control group was not statistically different at baseline, (5) at least one of the evaluated groups was based on FTS, (6) included articles provide sufficient data for analysis, (7) language was limited to English or Chinese, and (8) the study was extracted from January 1, 2015, to August 1, 2020.

The following exclusion criteria were used: (1) nonclinical trials, case reports or series; (2) animal experiments; (3) semi-randomized controlled trials or non-randomized trials; and (4) articles with incorrect or incomplete data, or articles whose data could not be extracted.

### Endpoints

The primary endpoints for this study were length of stay (LOS), Harris hip joint function score, VAS, satisfaction.

The secondary endpoints for this study were the leaving bed time.

The postoperative complication endpoints included respiratory system infection, urinary system infection, venous thrombus embolism (VTE), pressure sore, incision infection, constipation, and prosthesis dislocation.

### Data extraction

The two authors independently reviewed the contents of the retrieval research. The primary endpoints were extracted by two authors and verified by a third author. The extracted data included the following main information: first author’s name, year of publication, sample size, sex ratio, average age, clinical diagnosis or operative type, FTS measures, follow-up time, and endpoints measured in each study. If the contents of the studies needed to be clarified, please contact the first author of the study. Disagreements were resolved through consensus or consultation with a third author.

### Risk-of-bias assessments

The quality of the methodology in included studies was independently evaluated by the two authors according to the Cochrane Risk of Bias criteria. Each quality item was divided into low risk, high risk, and no obvious risk. The seven items used to estimate bias in each trial included randomization sequence generation, allocation concealment, blinding of participants and personnel, blinding of outcome assessment, incomplete outcome data, selective reporting, and other biases.

### Statistical analysis

Stata (version 12.0, Stata Corp, College Station, Texas) was used to analyse and pool the individual research results. Pooled results were recorded as risk ratios (RR) Standard mean difference (SMD) and 95% confidence intervals (CI) with two-sided *P* values. *P* values <0.05 were considered to be statistically significant. Heterogeneity was evaluated using the *I*^2^ test. The heterogeneity was considered to be small when *I*^2^<50% and substantial when *I*^2^>50%. The fixed effect model was used when *I*^2^ < 50%, while the random effect model was used when *I*^2^>50%. A funnel plot was generated to examine the publication bias and to explore the sources of heterogeneity if more than ten studies were included to assess this endpoint.

## Results

### Studies retrieved and characteristics

**Fig. 1:**
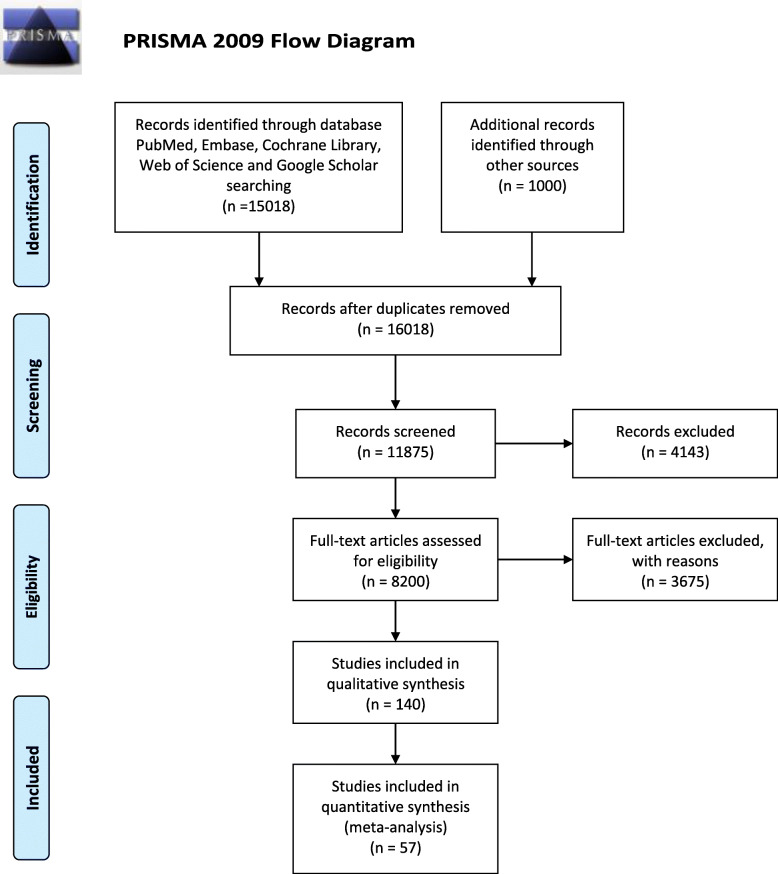
Flow diagram of the study selection process. CNKI China national knowledge infrastructure, VIP China Science and Technology Journal Database

A total of 16018 relevant studies were enrolled according to PRISMA guidelines. The titles and abstracts of the studies were screened to exclude irrelevant studies. Then, we further eliminated the unfit studies by reading the full text of the articles. Finally, 57 studies [21–77] were included according to the inclusion and exclusion criteria and they had a total of 8886 patients as shown in Fig. 1. In general, 4448 patients (50.06 %) were randomized to experimental group whereas 4438 patients (49.94 %) were randomized to control group. All studies included in this meta-analysis were RCTs. The basic characteristics of the individuals from the trials are described in Table 1.

**Table 1:** Characteristics of studies included in the meta-analysis. FTS fast track surgery, NA not applicable

Author	Publication year	Characteristics of study population						Hip fracture surgery		FTS measures	Follow-up	Endpoints
		Sample size (*n*)		Age (years)		Women, No. (%)						
		E	C	E	C	E	C	E	C			
Li [21]	2016	60	60	72.05±6.37	70.43±5.83	21 (35.00)	24 (40.00)	Total hip arthroplasty; hemiarthroplasty	Total hip arthroplasty; hemiarthroplasty	1, 2, 3, 4, 5, 7, 8	8 weeks	LOS, Harris hip joint function score, satisfaction, VTE, dislocation of prosthesis
Tian [22]	2016	40	40	NA	NA	NA	NA	Total hip arthroplasty	Total hip arthroplasty	1, 2, 8	6 months	LOS, respiratory, incision infection
Xu [23]	2016	55	55	73.58±5.27	73.45±5.46	32 (58.18)	34 (61.82)	Hip arthroplasty; internal fixation	Hip arthroplasty; internal fixation	1, 2, 3, 4, 5, 6, 7, 8	6 months	LOS, Harris hip joint function score, respiratory, urinary tract infection, VTE, pressure sores, dislocation of prosthesis
Yang [24]	2016	126	132	64.2±9.4	66.3±8.6	70 (27.1)	67 (26.0)	Unilateral hip arthroplasty	Unilateral hip arthroplasty	1, 2, 6, 7, 8	3 months	LOS, VTE, dislocation of prosthesis
Zhang [25]	2016	56	52	73.91±7.18	74.28±6.85	38 (67.86)	37 (71.15)	Hip arthroplasty; internal fixation	Hip arthroplasty; internal fixation	1, 4, 6, 7, 8	NA	LOS, Harris hip joint function score, VAS, respiratory, urinary tract infection, VTE, pressure sores
Chen 1 [26]	2017	106	92	68.5±4.1	67.7±4.3	61 (57.55)	50 (54.35)	NA	NA	1, 2, 4, 5, 6, 7, 8	NA	LOS, satisfaction, cost, VTE, pressure sores, constipation
Chen 2 [27]	2017	40	40	NA	NA	NA	NA	Unilateral hip arthroplasty	Unilateral hip arthroplasty	1, 7, 8	3d, 7d, 1 month, 3 months	Harris hip joint function score, VAS, respiratory, VTE, dislocation of prosthesis
Fu [28]	2017	40	40	NA	NA	NA	NA	NA	NA	1, 2, 4, 6, 7, 8	2 weeks, 1 month, 3 months, 6 months	Harris hip joint function score, VAS, respiratory, urinary tract infection, VTE, pressure sores
Li [29]	2017	45	45	NA	NA	NA	NA	Hip arthroplasty	Hip arthroplasty	2, 4, 8	8 weeks	Harris hip joint function score
Liu [30]	2017	41	41	53.08±2.07	56.02±1.85	12(29.27)	14(34.15)	Hip arthroplasty	Hip arthroplasty	3, 4, 6, 8	6 months	LOS, respiratory, urinary tract infection, VTE, incision infection
Wan [31]	2017	43	43	NA	NA	NA	NA	Hip arthroplasty	Hip arthroplasty	1, 4, 7, 8	NA	LOS
Wei [32]	2017	50	50	74.1±6.3	73.2±5.2	NA	NA	NA	NA	1, 2, 3, 4, 5, 6, 7, 8	NA	LOS, satisfaction, VTE
Yu [33]	2017	40	40	NA	NA	NA	NA	Hip arthroplasty	Hip arthroplasty	3, 4, 5, 6, 7, 8	6 months	LOS, Harris hip joint function score, cost, respiratory, urinary tract infection, VTE, incision infection
Zhang [34]	2017	43	43	70.13±3.75	69.38±3.46	18 (41.86)	17 (39.53)	Hip arthroplasty	Hip arthroplasty	1, 3, 4, 7, 8	NA	LOS, Harris hip joint function score, VTE, pressure sores
Zou [35]	2017	40	40	NA	NA	NA	NA	NA	NA	8	NA	LOS, Harris hip joint function score, respiratory, urinary tract infection, VTE
Ding [36]	2018	45	45	66.2±3.5	65.8±3.4	20 (44.44)	19 (42.22)	Total hip arthroplasty, unilateral hip arthroplasty	Total hip arthroplasty, unilateral hip arthroplasty	1, 2, 4, 5, 7, 8	NA	LOS, cost, respiratory, urinary tract infection, VTE, constipation
Jin [37]	2018	40	40	70.3±4.5	69.3±4.2	17 (42.50)	18 (45.00)	Hip arthroplasty, reduction of hip fracture	Hip arthroplasty, reduction of hip fracture	4, 7, 8	NA	LOS, VAS
Li 1 [38]	2018	41	41	63.5±3.5	64.5±4.5	18 (43.90)	19 (46.34)	Hip arthroplasty	Hip arthroplasty	1, 4, 6, 7, 8	NA	Harris hip joint function score, VTE
Li 2 [39]	2018	60	60	78.77±7.62	76.33±8.75	32 (53.33)	29 (48.33)	NA	NA	3, 4, 6, 8	NA	LOS, respiratory, urinary tract infection, VTE, pressure sores
Liu [40]	2018	53	53	72.42±2.29	72.39±2.25	17 (32.08)	20 (37.74)	NA	NA	1, 4, 7, 8	NA	VAS
Qian [41]	2018	41	41	81.35±4.77	81.21±4.46	17 (41.46)	18 (43.90)	Reduction of hip fracture, hip arthroplasty	Reduction of hip fracture, hip arthroplasty	1, 4, 7, 8	5 months	Harris hip joint function score, VAS
Wang [42]	2018	47	46	79.47±8.36	79.58±8.42	22 (46.81)	21 (45.65)	Hip arthroplasty	Hip arthroplasty	1, 4, 5, 7, 8	NA	LOS, satisfaction, the leaving bed time, urinary tract infection, VTE, incision infection
Yang 1 [43]	2018	50	50	58.52±8.53	57.96±8.41	25 (50.00)	24 (48.00)	Hip arthroplasty	Hip arthroplasty	1, 2, 3, 5, 7, 8	NA	LOS, Harris hip joint function score, VTE, pressure sores
Yang [44]	2018	40	40	57.4 ±11.3	58.3 ±12.9	22 (55.00)	19 (47.50)	Hip arthroplasty	Hip arthroplasty	1, 2, 4, 7, 8	1d, 3d, 7d	VAS, the leaving bed time, VTE, incision infection
You [45]	2018	100	100	86±5.1	82±5.3	40 (40.00)	42 (42.00)	PFNA internal fixation	PFNA internal fixation	1, 2, 3, 4, 5, 6, 7, 8	NA	LOS, satisfaction, cost
Zang [46]	2018	40	40	71.5±1.5	70.5±3.0	19 (47.50)	20 (50.00)	Femoral head replacement	Femoral head replacement	1, 2, 4, 7, 8	18 months	Satisfaction
Zhai [47]	2018	40	40	73.45±2.54	72.99±3.14	19 (47.50)	22 (55.00)	NA	NA	1, 5, 7, 8	NA	Satisfaction, VTE, pressure sores
Zheng [48]	2018	45	45	71.16±5.05	71.08±5.07	19 (42.22)	20 (44.44)	Hip arthroplasty	Hip arthroplasty	4, 6, 7, 8	6 months	LOS, Harris hip joint function score, cost, VTE
Zuo [49]	2018	40	41	72.36±10.48	71.72±10.2 4	19 (47.50)	18 (43.90)	Hip arthroplasty	Hip arthroplasty	1, 4, 5, 6, 8	3 months	Harris hip joint function score, respiratory, VTE
Bai [50]	2019	64	64	NA	NA	NA	NA	Total hip arthroplasty, unilateral hip arthroplasty	Total hip arthroplasty, unilateral hip arthroplasty	1, 2, 4, 5, 7, 8	3 months	Harris hip joint function score, VTE, pressure sores, dislocation of prosthesis
Bao [51]	2019	49	48	NA	NA	NA	NA	NA	NA	2, 4, 5, 6, 7, 8	6 months	Harris hip joint function score, VAS, respiratory, urinary tract infection, VTE, pressure sores
Chen [52]	2019	44	43	68.21±6.44	65.58±6.34	29 (65.91)	28 (65.12)	Hip arthroplasty	Hip arthroplasty	1, 3, 4, 7, 8	NA	LOS, satisfaction, the leaving bed time, cost
Fusco [53]	2019	40	40	NA	NA	0	0	Hip arthroplasty	Hip arthroplasty	8	12 months	Cost
Guo 1 [54]	2019	51	51	72.6±3.5	72.3±3.4	18 (35.29)	21 (41.18)	Hip arthroplasty	Hip arthroplasty	1, 2, 3, 4, 5, 7, 8	7d	LOS, respiratory, VTE, incision infection
Guo 2 [55]	2019	42	42	82.24±4.58	82.29±4.73	17 (40.48)	18 (42.86)	NA	NA	1, 4, 7, 8	5 months	Harris hip joint function score
He [56]	2019	43	43	83.85±3.79	82.47±3.18	19 (44.19)	21 (48.84)	Reduction of hip fracture, hip arthroplasty	Reduction of hip fracture, hip arthroplasty	1, 4, 8	NA	LOS, Harris hip joint function score, VAS, cost, urinary tract infection, VTE, pressure sores
Huang [57]	2019	40	40	NA	NA	NA	NA	Hip arthroplasty	Hip arthroplasty	1, 8	NA	LOS
Jia [58]	2019	60	60	NA	NA	NA	NA	Hip arthroplasty	Hip arthroplasty	1, 2, 4, 5, 6, 8	NA	LOS, VAS, satisfaction, the leaving bed time, VTE, pressure sores
Jiang [59]	2019	43	43	46.5±2.4	41.5±5.7	20 (46.51)	24 (55.81)	NA	NA	1, 4, 7, 8	5 months	Satisfaction
Jin 1 [60]	2019	43	43	68.5±5.6	66.4±5.6	23 (53.49)	21 (48.84)	NA	NA	1, 4, 5, 8	NA	LOS, satisfaction, cost
Jin 2 [61]	2019	132	146	73.5±7.6	71.7±5.2	103 (78.03)	101 (69.18)	Hip arthroplasty	Hip arthroplasty	4, 5, 6, 7, 8	1d, 2d, 1 week	The leaving bed time, respiratory, urinary tract infection, VTE, incision infection
Li [62]	2019	40	40	62.30±10.40	62.25±10.34	18 (45.00)	17 (42.50)	Hip arthroplasty	Hip arthroplasty	1, 2, 3, 4, 6, 7, 8	1 month, 3 months	LOS, cost
Liang [63]	2019	53	53	76.7±2.4	77.9±2.7	24 (45.28)	21 (39.63)	NA	NA	1, 4, 5, 6, 7, 8	3 months	Harris hip joint function score, respiratory, urinary tract infection, VTE, pressure sores
Liu [64]	2019	60	60	81.9±4.9	81.6±5.2	17 (28.33)	13 (21.67)	NA	NA	3, 5, 6, 8	NA	LOS
Sun [65]	2019	45	45	69.24±2.89	69.78±2.82	13 (28.89)	14 (31.11)	NA	NA	1, 3, 4, 5, 6, 7, 8	NA	Harris hip joint function score, satisfaction, respiratory, urinary tract infection, VTE, pressure sores
Xiao [66]	2019	40	40	72.11±4.35	71.39±4.15	17 (42.50)	19 (47.50)	Unilateral hip arthroplasty	Unilateral hip arthroplasty	1, 4, 8	NA	VTE, constipation
Yang [67]	2019	56	56	70.24±17.76	71.57±17.43	33 (58.93)	32 (57.14)	Hip arthroplasty; Internal fixation	Hip arthroplasty; Internal fixation	1, 7, 8	NA	LOS
Yu [68]	2019	66	60	73.94±3.87	73.77±4.25	40 (60.60)	37 (61.67)	Hip arthroplasty; Internal fixation	Hip arthroplasty; Internal fixation	1, 2, 4, 5, 7, 8	3 months	LOS, satisfaction, respiratory, urinary tract infection, VTE, dislocation of prosthesis
Zhang [69]	2019	49	49	73.14±2.28	73.75±2.54	20 (40.82)	22 (44.90)	Hip arthroplasty	Hip arthroplasty	4, 6, 8	NA	LOS, satisfaction
Zhu [70]	2019	90	90	72.1±8.3	72.4±8.6	52 (57.78)	50 (55.56)	NA	NA	1, 4, 6, 7, 8	6 months	Harris hip joint function score, VAS, respiratory, urinary tract infection, VTE, pressure sores, incision infection
Borges [71]	2020	1487	1483	NA	NA	1031 (69.33)	1022 (68.91)	Open reduction and internal fixation, hip arthroplasty	Open reduction and internal fixation, hip arthroplasty	NA	NA	Respiratory, VTE, pressure sores
Du [72]	2020	50	50	83.1±2.4	84.6±2.8	38 (76.00)	34 (68.00)	Total hip arthroplasty	Total hip arthroplasty	1, 4, 5, 6, 7, 8	6 months	Harris hip joint function score, VAS, the leaving bed time, urinary tract infection, VTE, pressure sores, dislocation of prosthesis
Ge [73]	2020	40	40	NA	NA	27 (67.50)	28 (70.00)	Hip arthroplasty	Hip arthroplasty	1, 3, 4, 5, 6, 7, 8	3 months	LOS, Harris hip joint function score, VTE, constipation
Jiang [74]	2020	40	40	NA	NA	NA	NA	Internal fixation	Internal fixation	8	2 weeks, 1 month, 3 months	Harris hip joint function score, respiratory, urinary tract infection, VTE, constipation
Liang [75]	2020	47	47	46.58±5.97	47.83±5.52	20 (42.55)	21 (44.68)	Hip arthroplasty	Hip arthroplasty	1, 3, 4, 7, 8	NA	LOS, satisfaction, the leaving bed time, cost
Yu [76]	2020	47	47	76.5±3.2	75.9±3.8	19 (40.43)	20 (42.55)	NA	NA	1, 3, 4, 6, 7	NA	LOS, VAS, satisfaction, the leaving bed time, respiratory, urinary tract infection, VTE, pressure sores
Zheng [77]	2020	80	80	59.66±5.26	59.63±5.14	33 (41.25)	35 (43.75)	Hip arthroplasty	Hip arthroplasty	1, 7, 8	1 month, 6 months	Harris hip joint function score

*FTS* fast track surgery, *NA* not applicable. FTS measures: (1) preoperative propaganda and education/psychological counseling, (2) the method of optimizing anesthesia, (3) simplification of routine intestinal preparation before operation, (4) perioperative nutrition management, (5) perioperative heat preservation, (6) rational use of drainage tube and catheter, (7) postoperative analgesia, and (8) early postoperative normative functional exercise

### Literature quality evaluation

The Cochrane Risk of Bias criteria was used to evaluate the quality of the retrieved studies by two authors. The included studies were all randomized controlled trials. Fifty-seven studies [21–77] described random sequence generation and allocation concealment. Four studies [53, 57, 66, 72] described blinding of participants and personnel. Four studies [53, 57, 66, 72] described blinding of outcome assessment. None of the studies described other biases. The literature quality score is shown in Table 2.

**Table 2:** Assessment of methodological quality of included studies

Author	Random allocation	Hidden distribution	Blind method	Incomplete Outcome Data	Selective reporting of results	Other bias	Quality grade
Li [21]	Randomized	No clear	No clear	Low	Low	Low	B
Tian [22]	Randomized	No clear	No clear	Low	Low	Low	C
Xu [23]	Randomized	No clear	No clear	Low	Low	Low	B
Yang [24]	Randomized	No clear	No clear	Low	Low	Low	B
Zhang [25]	Randomized	No clear	No clear	Low	Low	Low	B
Chen 1 [26]	Randomized	No clear	No clear	Low	Low	Low	B
Chen 2 [27]	Randomized	No clear	No clear	Low	Low	Low	C
Fu [28]	Randomized	No clear	No clear	Low	Low	Low	C
Li [29]	Randomized	No clear	No clear	Low	Low	Low	C
Liu [30]	Randomized	No clear	No clear	Low	Low	Low	B
Wan [31]	Randomized	No clear	No clear	Low	Low	Low	C
Wei [32]	Randomized	No clear	No clear	Low	Low	Low	B
Yu [33]	Randomized	No clear	No clear	Low	Low	Low	C
Zhang [34]	Randomized	No clear	No clear	Low	Low	Low	B
Zou [35]	Randomized	No clear	No clear	Low	Low	Low	C
Ding [36]	Randomized	No clear	No clear	Low	Low	Low	B
Jin [37]	Randomized	No clear	No clear	Low	Low	Low	B
Li 1 [38]	Randomized	No clear	No clear	Low	Low	Low	B
Li 2 [39]	Randomized	No clear	No clear	Low	Low	Low	B
Liu [40]	Randomized	No clear	No clear	Low	Low	Low	B
Qian [41]	Randomized	No clear	No clear	Low	Low	Low	B
Wang [42]	Randomized	No clear	No clear	Low	Low	Low	B
Yang 1 [43]	Randomized	No clear	No clear	Low	Low	Low	B
Yang [44]	Randomized	No clear	No clear	Low	Low	Low	B
You [45]	Randomized	No clear	No clear	Low	Low	Low	B
Zang [46]	Randomized	No clear	No clear	Low	Low	Low	B
Zhai [47]	Randomized	No clear	No clear	Low	Low	Low	B
Zheng [48]	Randomized	No clear	No clear	Low	Low	Low	B
Zuo [49]	Randomized	No clear	No clear	Low	Low	Low	B
Bai [50]	Randomized	No clear	No clear	Low	Low	Low	C
Bao [51]	Randomized	No clear	No clear	Low	Low	Low	C
Chen [52]	Randomized	No clear	No clear	Low	Low	Low	B
Fusco [53]	Randomized	No clear	Double-Blind	Low	Low	Low	B
Guo 1 [54]	Randomized	No clear	No clear	Low	Low	Low	B
Guo 2 [55]	Randomized	No clear	No clear	Low	Low	Low	B
He [56]	Randomized	No clear	No clear	Low	Low	Low	B
Huang [57]	Randomized	No clear	Double-Blind	Low	Low	Low	B
Jia [58]	Randomized	No clear	No clear	Low	Low	Low	C
Jiang [59]	Randomized	No clear	No clear	Low	Low	Low	B
Jin 1 [60]	Randomized	No clear	No clear	Low	Low	Low	B
Jin 2 [61]	Randomized	No clear	No clear	Low	Low	Low	B
Li [62]	Randomized	No clear	No clear	Low	Low	Low	B
Liang [63]	Randomized	No clear	No clear	Low	Low	Low	B
Liu [64]	Randomized	No clear	No clear	Low	Low	Low	B
Sun [65]	Randomized	No clear	No clear	Low	Low	Low	B
Xiao [66]	Randomized	No clear	Double-Blind	Low	Low	Low	A
Yang [67]	Randomized	No clear	No clear	Low	Low	Low	B
Yu [68]	Randomized	No clear	No clear	Low	Low	Low	B
Zhang [69]	Randomized	No clear	No clear	Low	Low	Low	B
Zhu [70]	Randomized	No clear	No clear	Low	Low	Low	B
Borges [71]	Randomized	No clear	No clear	Low	Low	Low	B
Du [72]	Randomized	No clear	Single-Blind	Low	Low	Low	A
Ge [73]	Randomized	No clear	No clear	Low	Low	Low	C
Jiang [74]	Randomized	No clear	No clear	Low	Low	Low	C
Liang [75]	Randomized	No clear	No clear	Low	Low	Low	B
Yu [76]	Randomized	No clear	No clear	Low	Low	Low	B
Zheng [77]	Randomized	No clear	No clear	Low	Low	Low	B

### Primary endpoints

#### Length of stay (LOS)

Thirty-three studies [21–26, 30–37, 39, 42, 43, 45, 48, 52, 54, 56–58, 60, 62, 64, 67–69, 73, 75, 76] reported length of stay (LOS). In total, 3526 patients were involved to evaluate LOS, wherein 1773 were assigned to experimental group and 1753 were assigned to control group. The result showed that patients’ LOS in experimental group was significantly less than that in control group (SMD: −1.94, 95% CI −2.29 to −1.59, *I*^2^=94.7%) as shown inFig. 2.

**Fig. 2:**
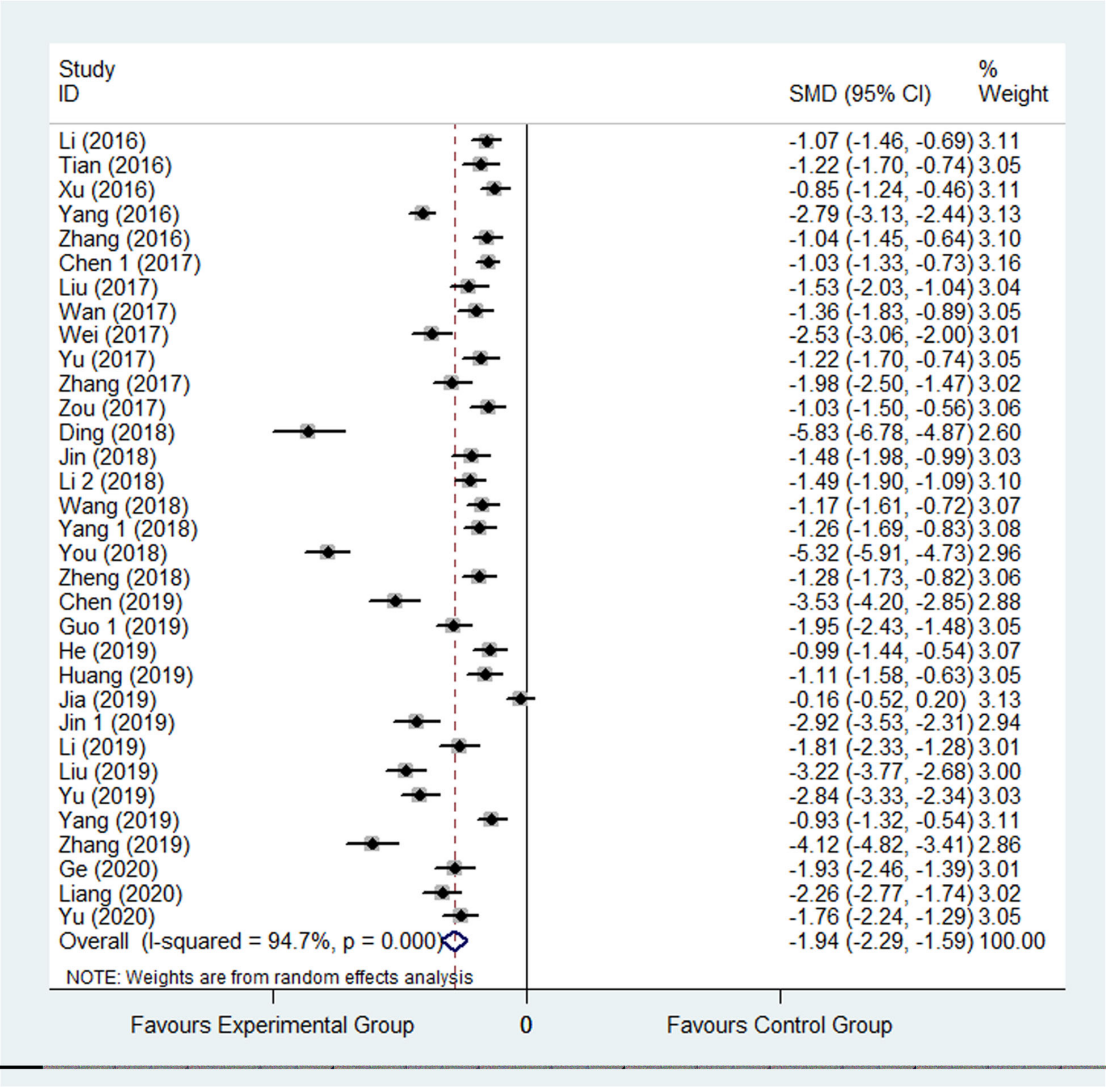
Comparison of LOS between the experimental group and the control group. SMD standardized mean difference, LOS length of stay

#### Harris Hip Joint Function Score

**Fig. 3:**
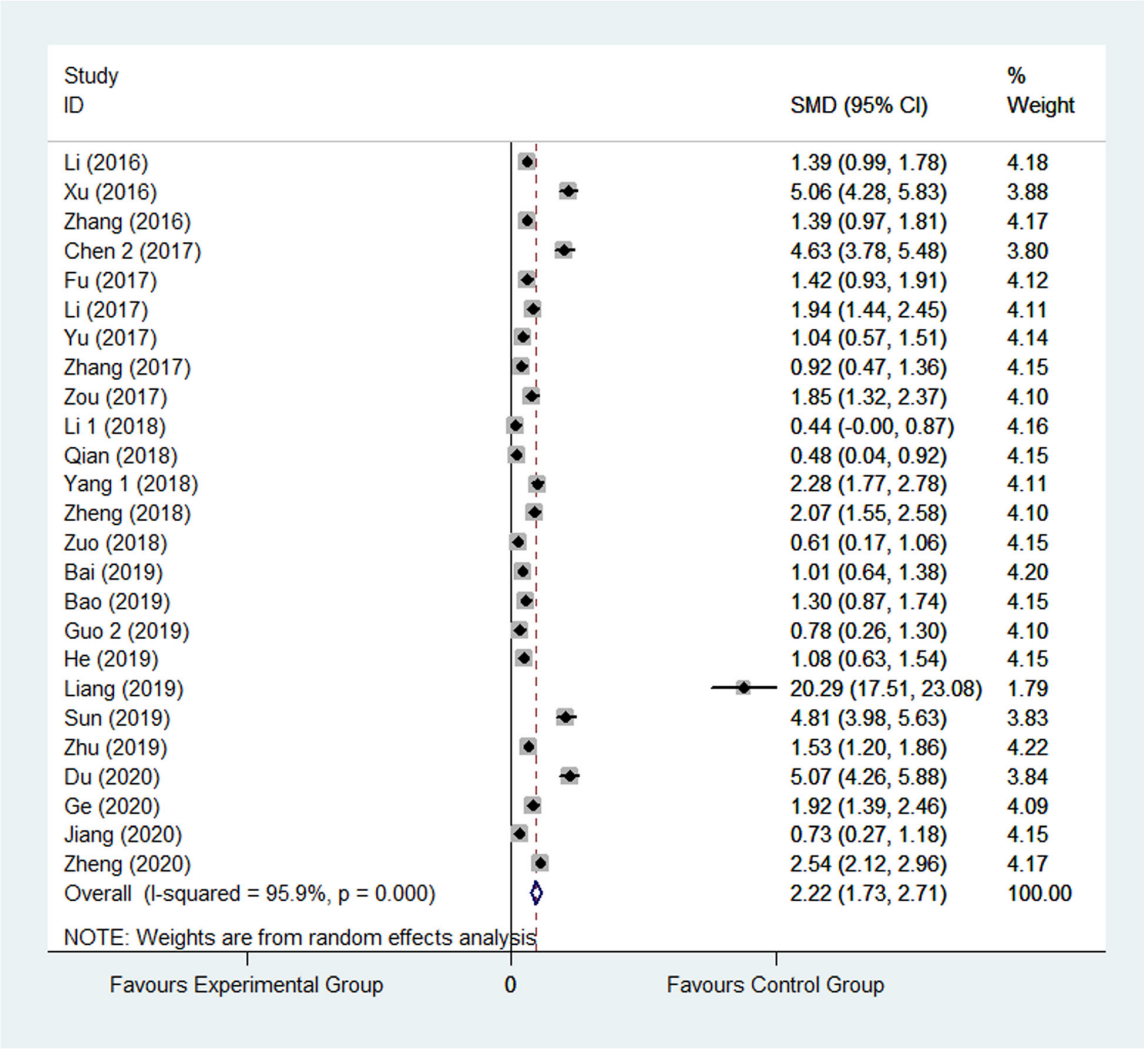
Comparison of Harris hip joint function score between the experimental group and the control group. SMD standardized mean difference

**Fig. 4:**
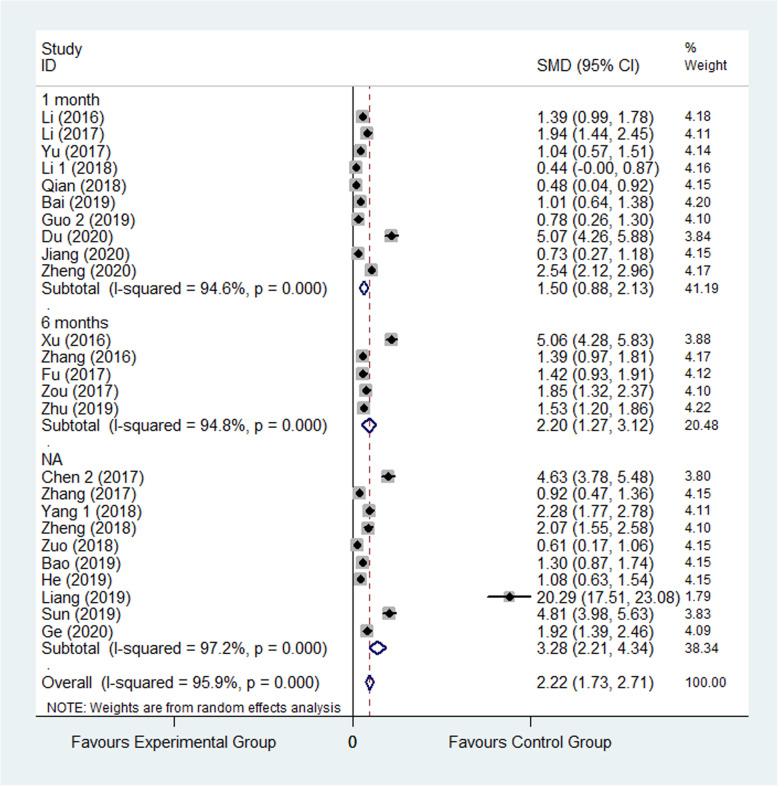
Comparison of Harris hip joint function score between the experimental group and the control group (subgroup analysis). SMD standardized mean difference

Harris hip joint function score scale is a widely used method to evaluate hip function. The higher the total score, the stronger the function. Harris hip joint function score was reported in 25 studies [21, 23, 25, 27–29, 33–35, 38, 41, 43, 48–51, 55, 56, 63, 65, 70, 72–74, 77]. 2441 patients were involved in all, wherein 1232 were assigned to the experimental group and 1209 were assigned to control group. The result showed that patients’ score in experimental group was significantly greater than that in control group (SMD: 2.22, 95% CI 1.73 to 2.71, *I*^2^=95.9%) as shown in Fig. 3. The random effect model was applied. Subgroup analysis was performed according to the follow-up period and divided into 1 month after surgery, 6 months after surgery, and not applicable three group. The result of follow-up time subgroup showed that patients’ Harris hip joint function score 6 months after surgery in experimental group was significantly higher than that in control group, though the not applicable group is the highest. (SMD: 1.50, 95% CI 0.88 to 2.13; SMD: 2.20, 95% CI 1.27 to 3.12; SD, 95% CI 2.21 to 4.34) as shown in Fig. 4.

#### VAS

**Fig. 5:**
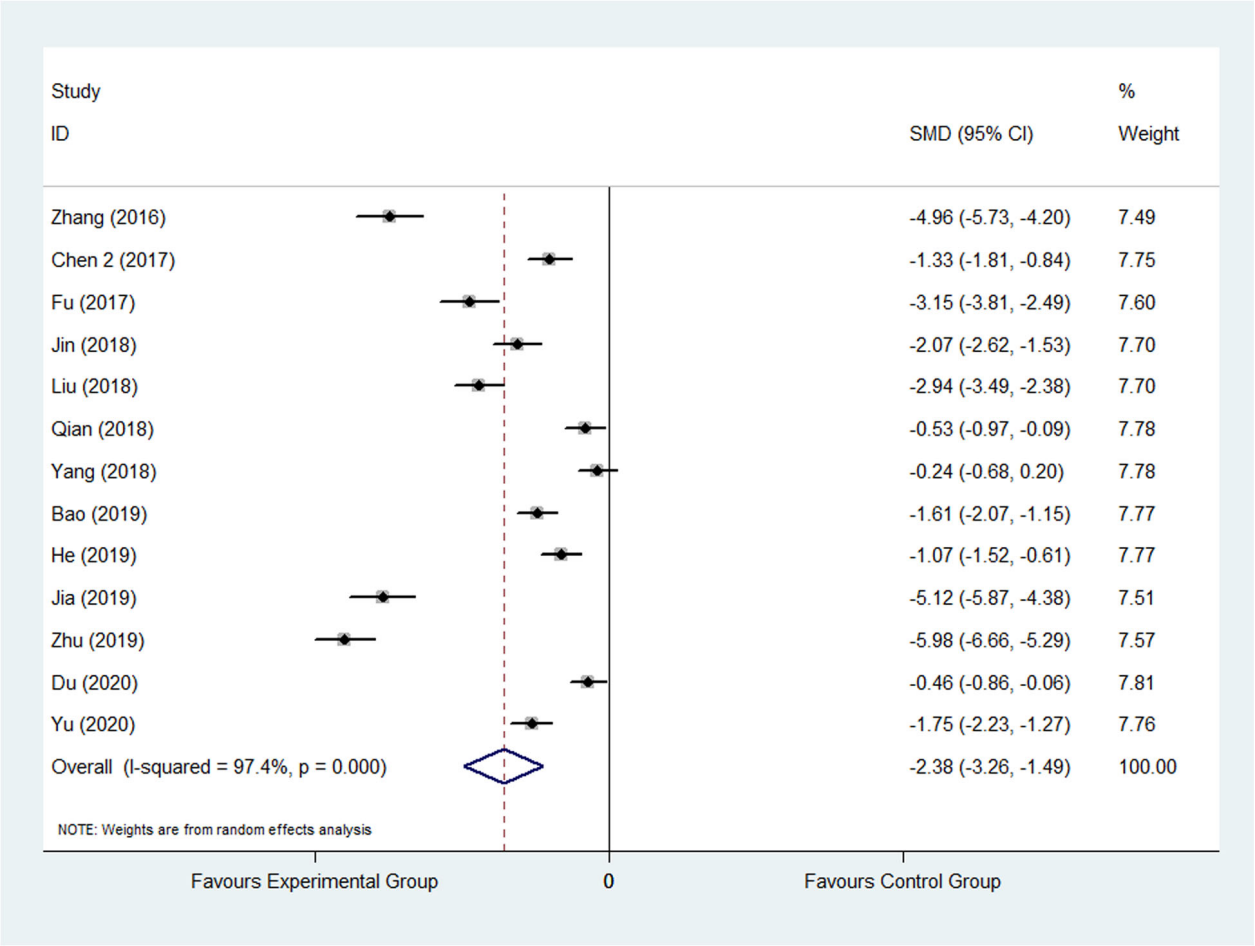
Comparison of VAS between the experimental group and the control group. SMD standardized mean difference, VAS visual analog scale

**Fig. 6:**
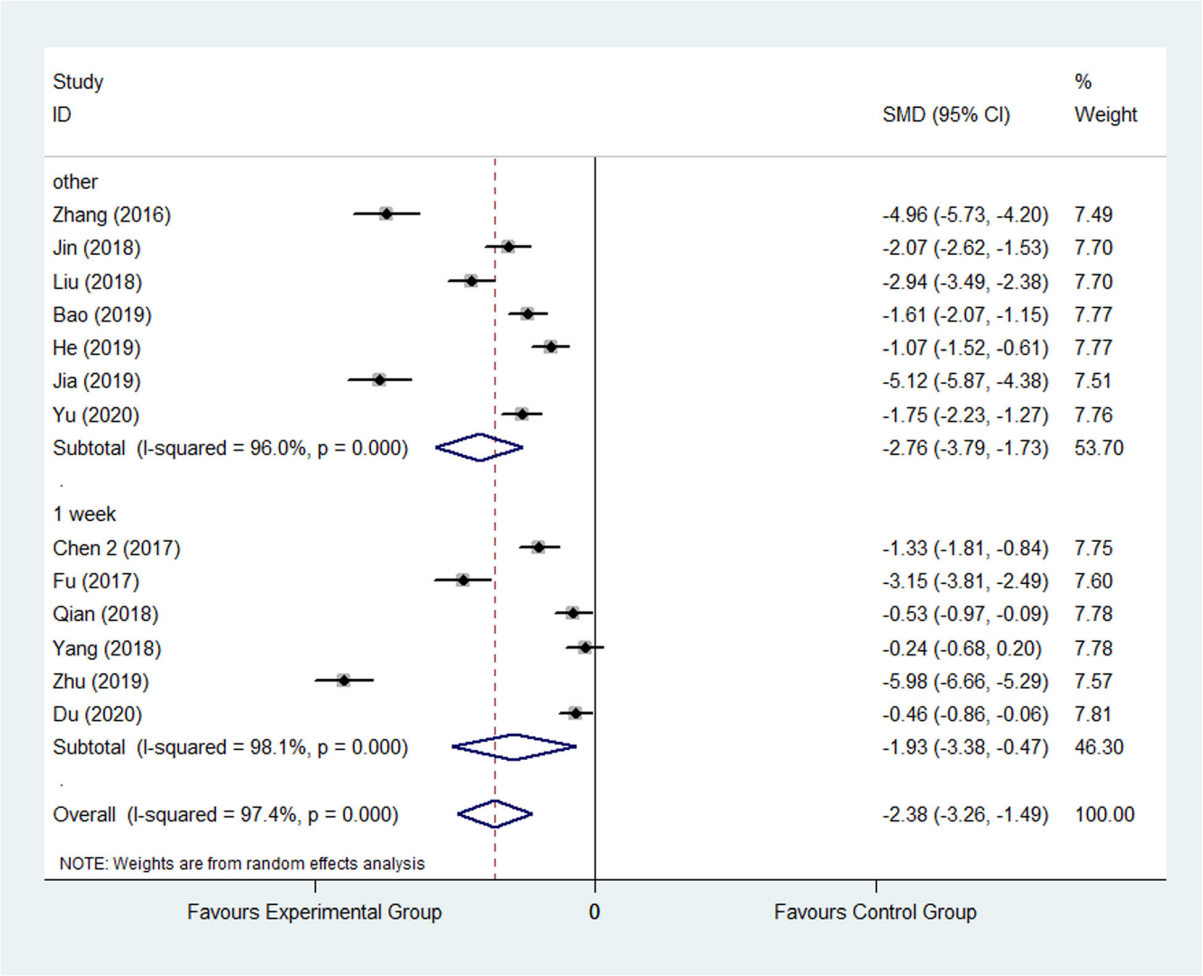
Comparison of VAS between the experimental group and the control group. (subgroup analysis). SMD standardized mean difference, VAS visual analog scale

Visual analog scale (VAS) was used to score the pain degree of patients, which the score was in direct proportion to the pain degree. It was reported by 13 studies [25, 27, 28, 37, 40, 41, 44, 51, 56, 58, 70, 72, 76] included 1293 patients, wherein 649 were assigned to the experimental group and 644 were assigned to control group. The result showed that FTS could reduce VAS significantly in experimental group than control group (SMD: −2.38, 95% CI −3.26 to −1.49, *I*^2^=97.4%) as shown in Fig. 5. The random effect model was applied. Subgroup analysis was performed according to the follow-up time and divided into 1 month after surgery and others’ group. The result of follow-up subgroup was showed that the VAS in experimental group was significantly less than that in control group in 1 month after surgery subgroup (SMD: −2.76, 95% CI −3.79 to −1.73; SMD: −1.93, 95% CI −3.38 to −0.47) as shown in Fig. 6.

#### Satisfaction

**Fig. 7:**
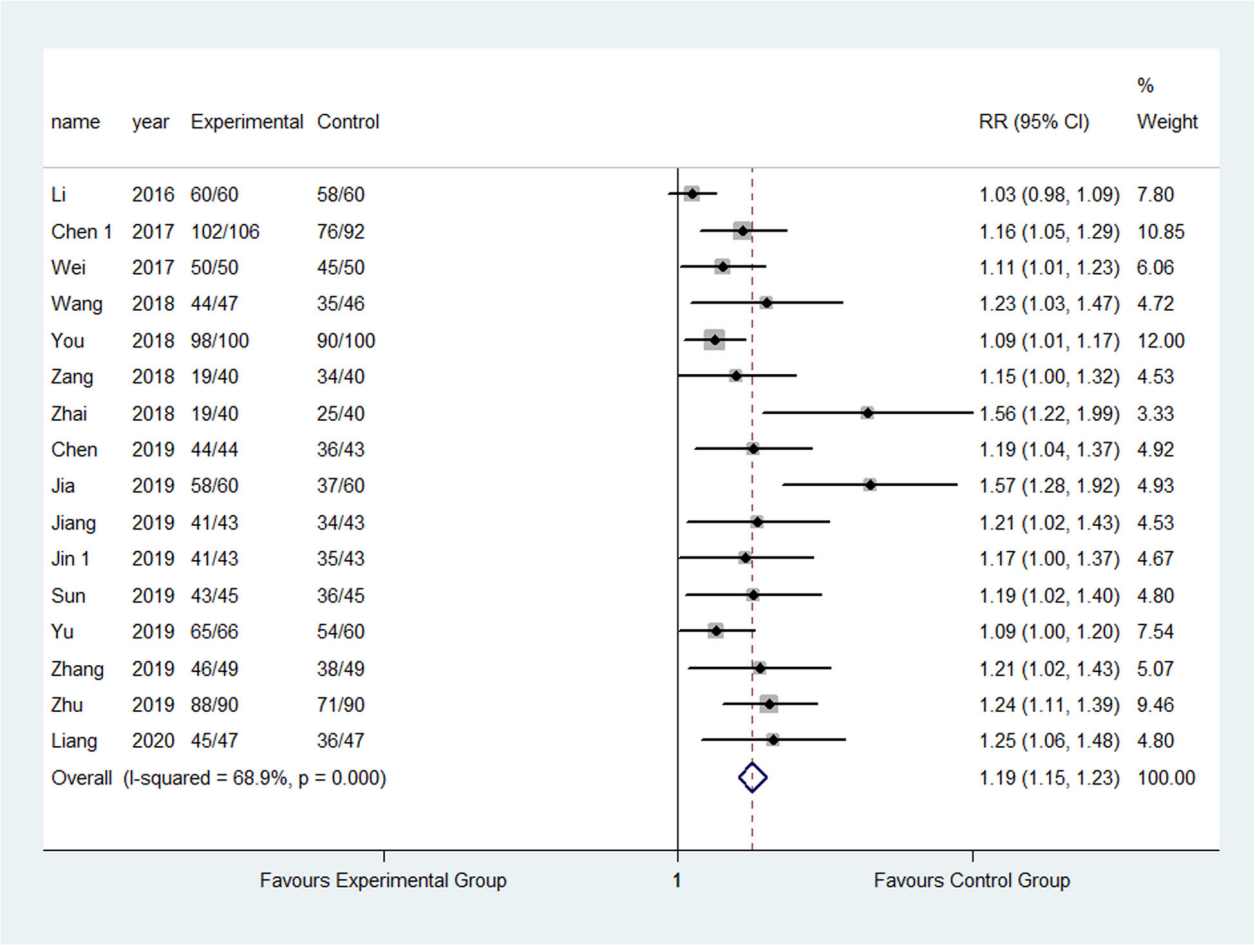
Comparison of satisfaction between the experimental group and the control group. RR risk ratio

Sixteen studies [21, 26, 32, 42, 45–47, 52, 58–60, 65, 68, 69, 75, 76] reported patients’ satisfaction about the FTS. In total, 903 out of 930 patients in experimental group satisfied with the FTS while 740 out of 908 patients in the control group satisfied with the standard care. The result showed that FTS care significantly raised the satisfaction compared to the control group (97.1% vs 81.0%) (RR: 1.19, 95% CI 1.15 to 1.23, *I*^2^=68.9%) as shown in Fig. 7.

#### Secondary endpoints

**Fig. 8:**
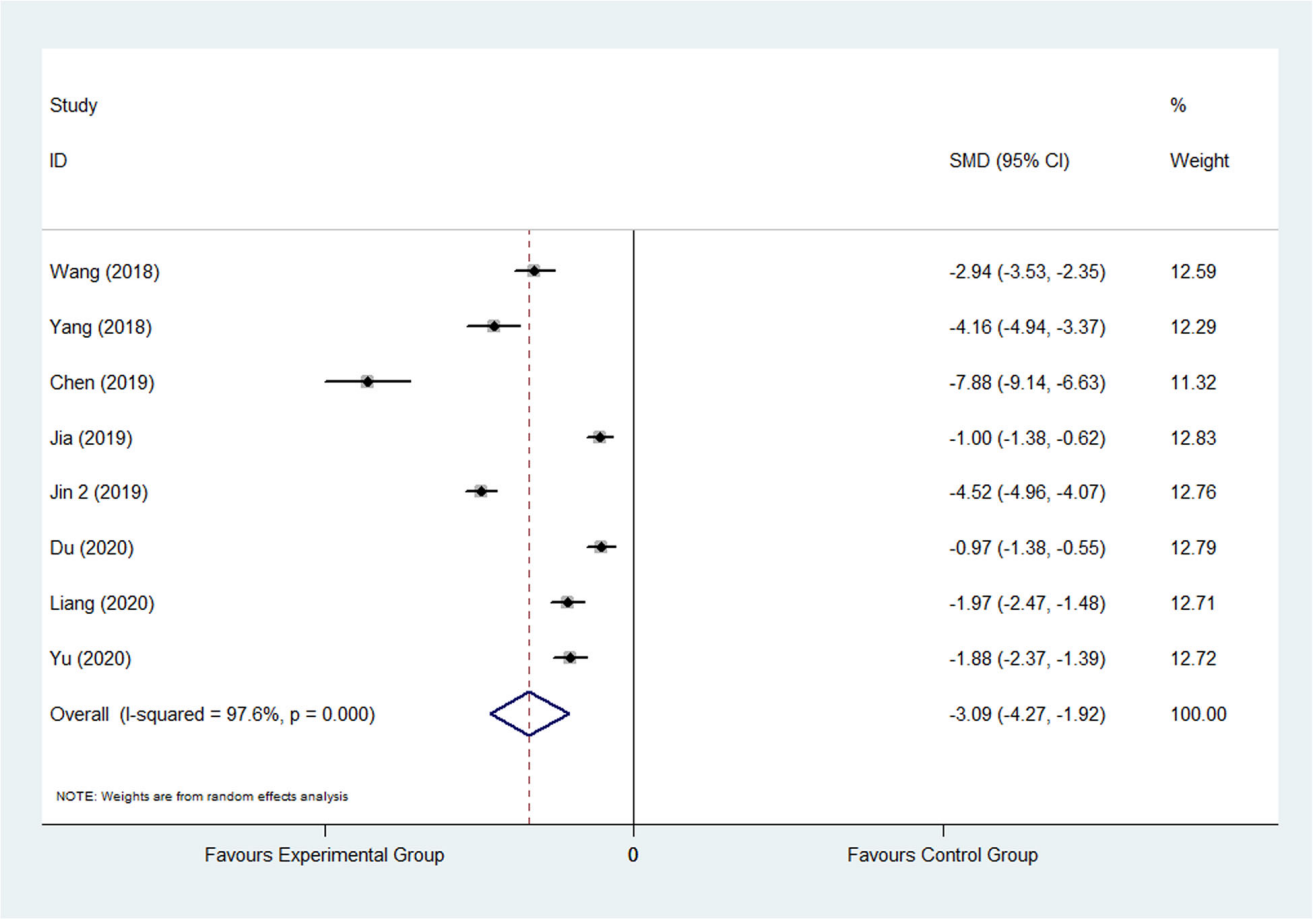
Comparison of the leaving bed time between the experimental group and the control group. SMD standardized mean difference

**Fig. 9:**
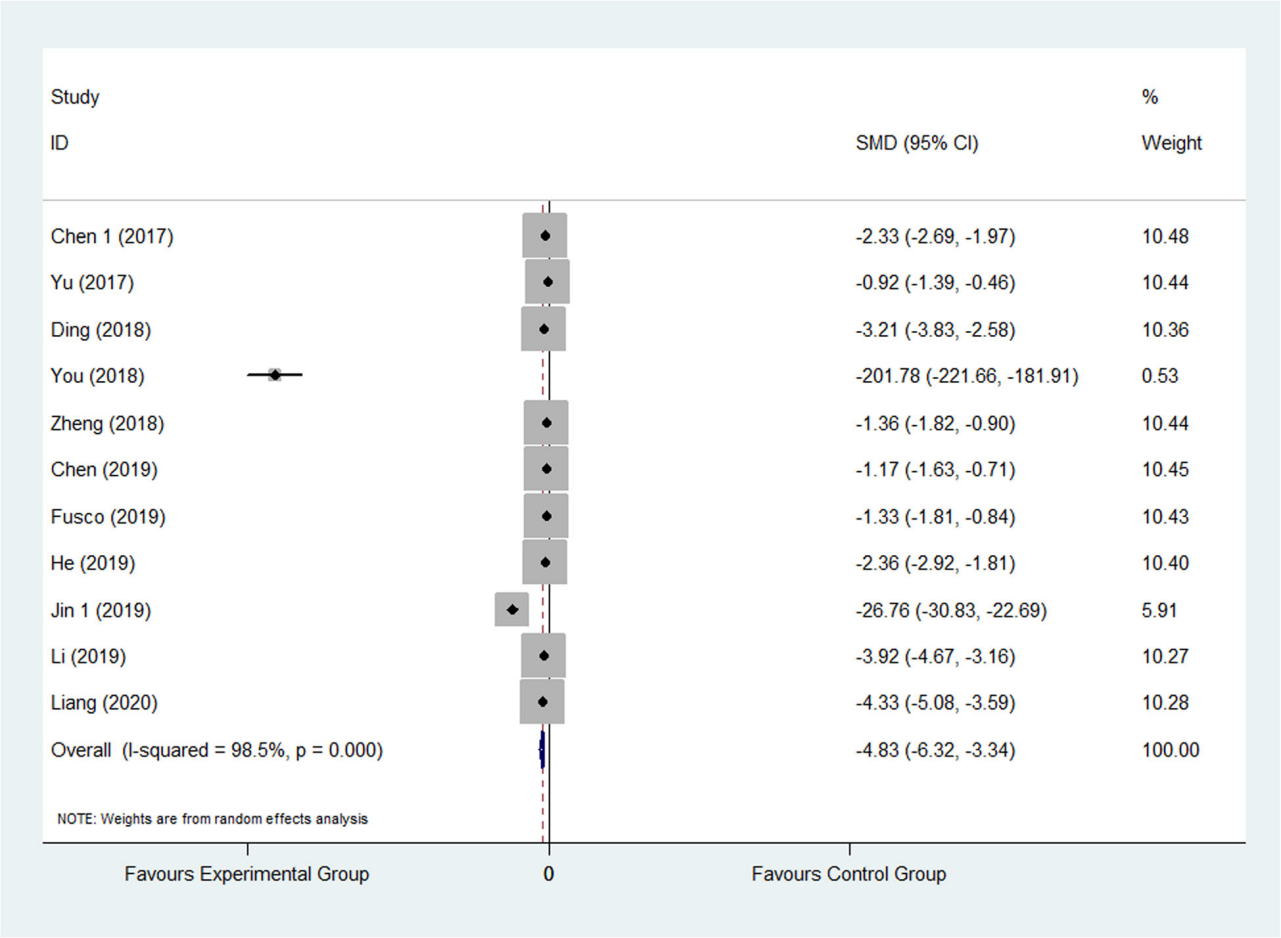
Comparison of cost between the experimental group and the control group. SMD standardized mean difference

The result showed that compared to the control group, FTS could significantly reduce the leaving bed time (SMD: −3.09, 95% CI −4.27 to −1.92, *I*^2^=97.6%) as shown in Fig. 8; it also could decrease the hospitalization costs (SMD: −4.83, 95% CI −6.32 to −3.34, *I*^2^=98.5%) as shown in Fig. 9.

**Fig. 10:**
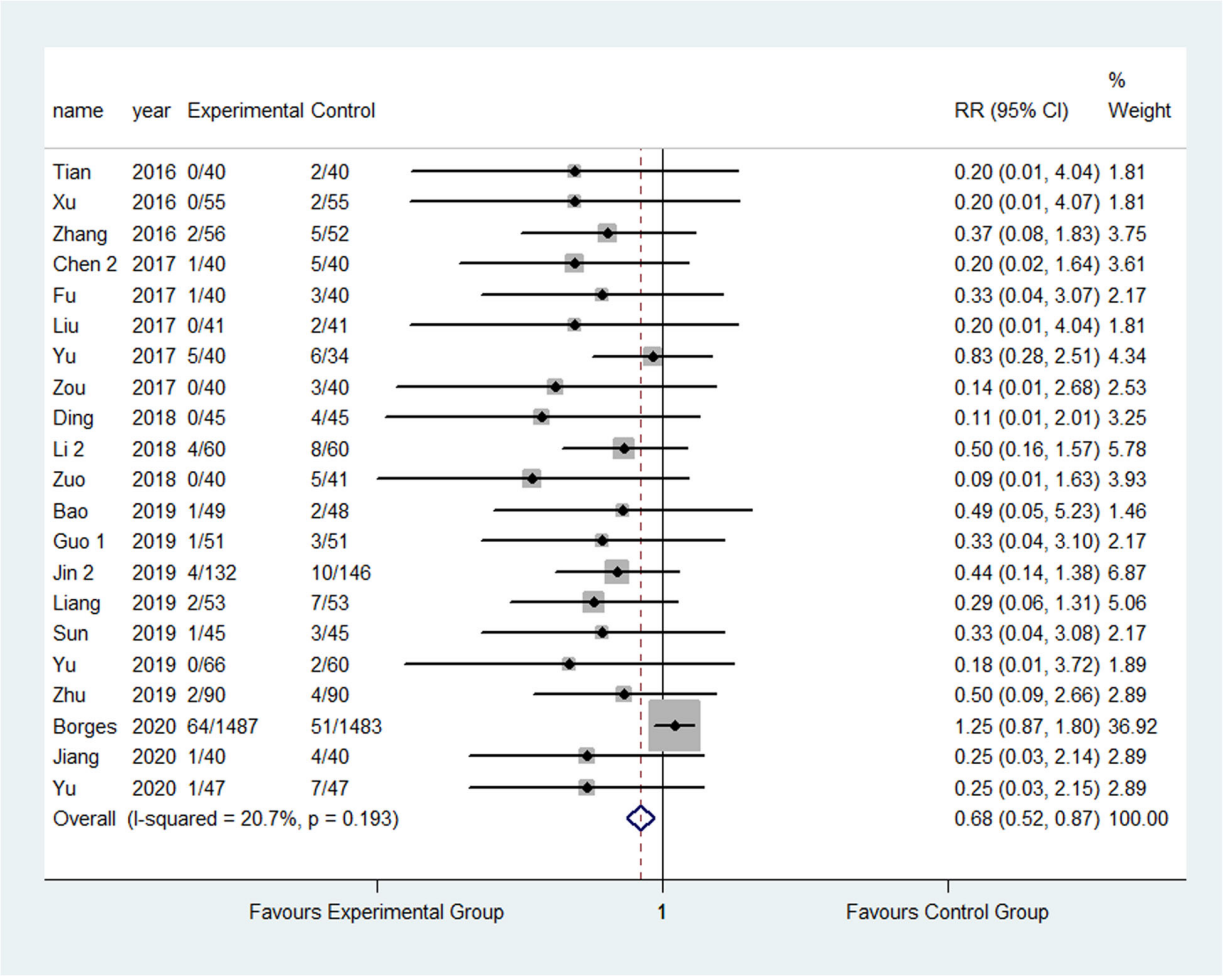
Incidence of respiratory infection between the experimental group and the control group. RR risk ratio

#### Postoperative complications endpoints

**Fig. 11:**
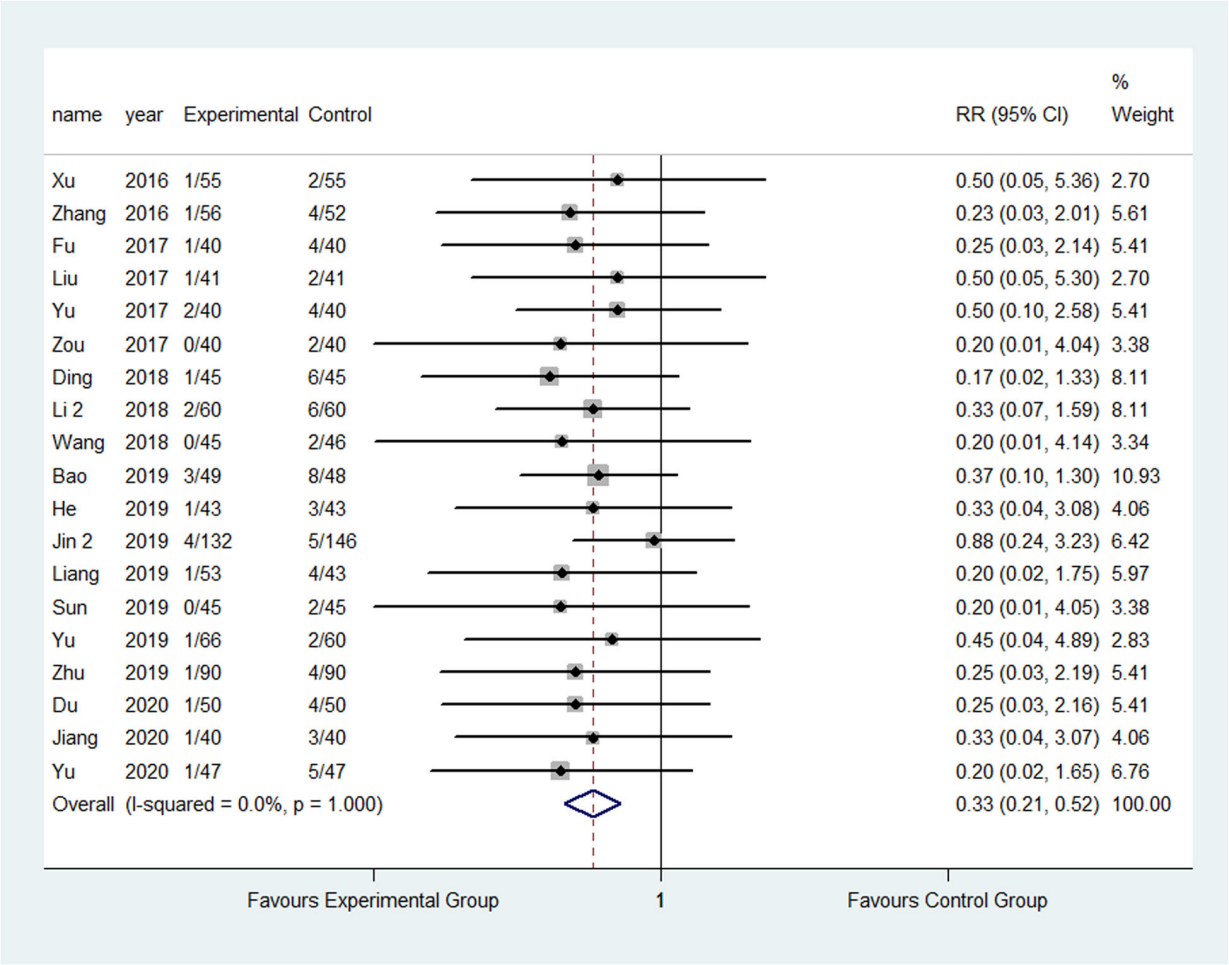
Incidence of urinary tract infection between the experimental group and the control group. RR risk ratio

**Fig. 12:**
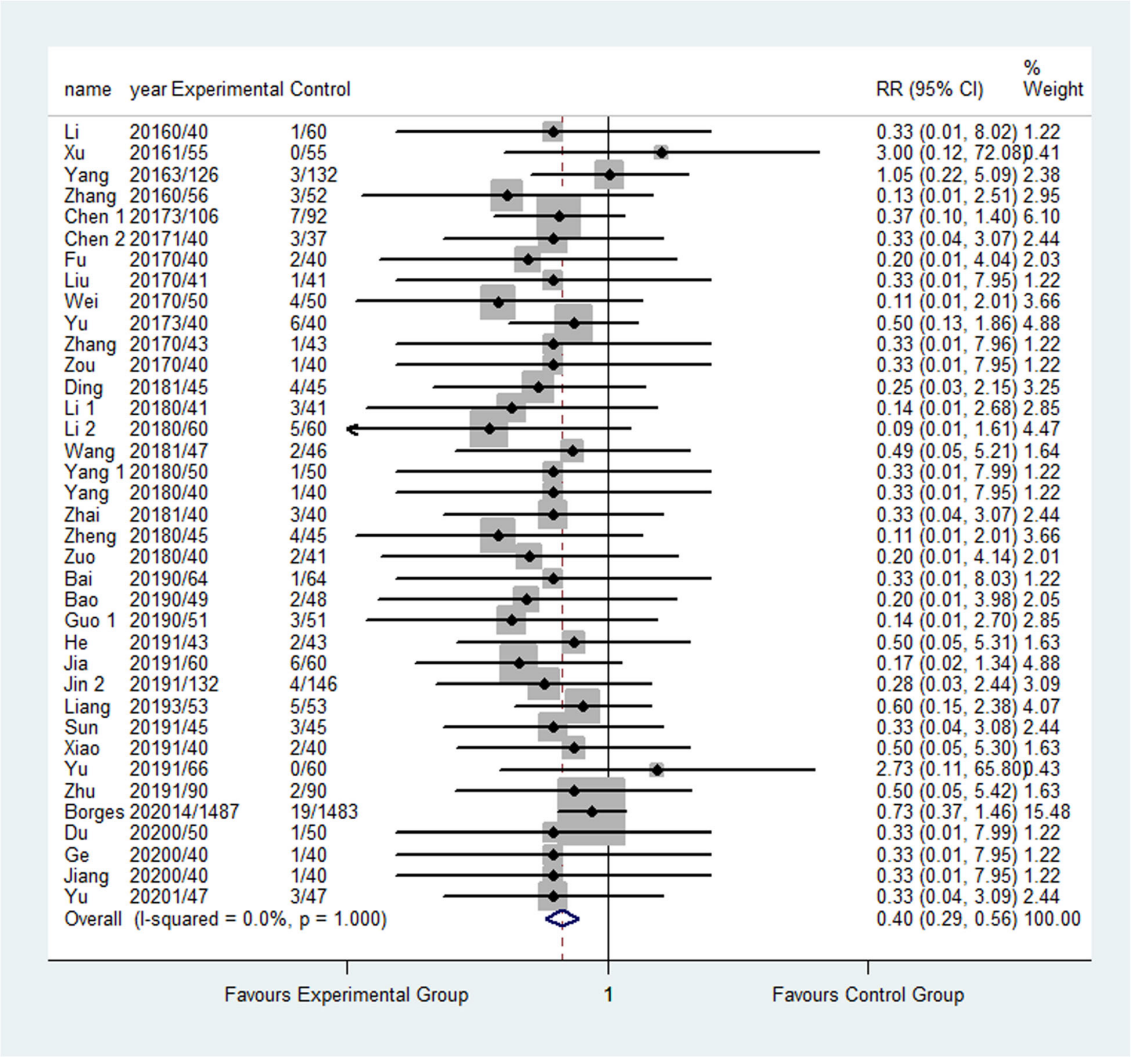
Incidence of VTE between the experimental group and the control group. RR risk ratio, VTE venous thrombus embolism

**Fig. 13:**
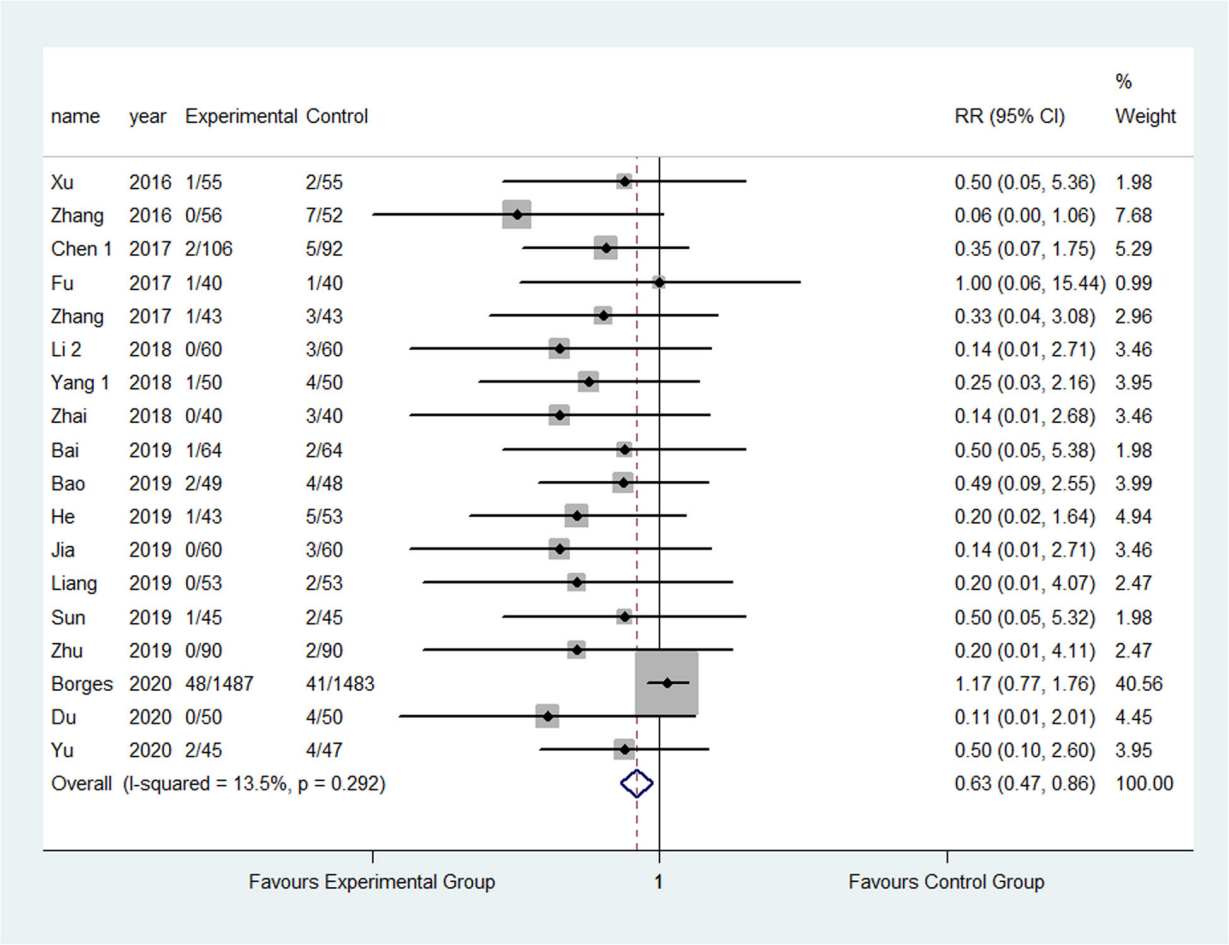
Incidence of pressure sores between the experimental group and the control group. RR risk ratio

**Fig. 14:**
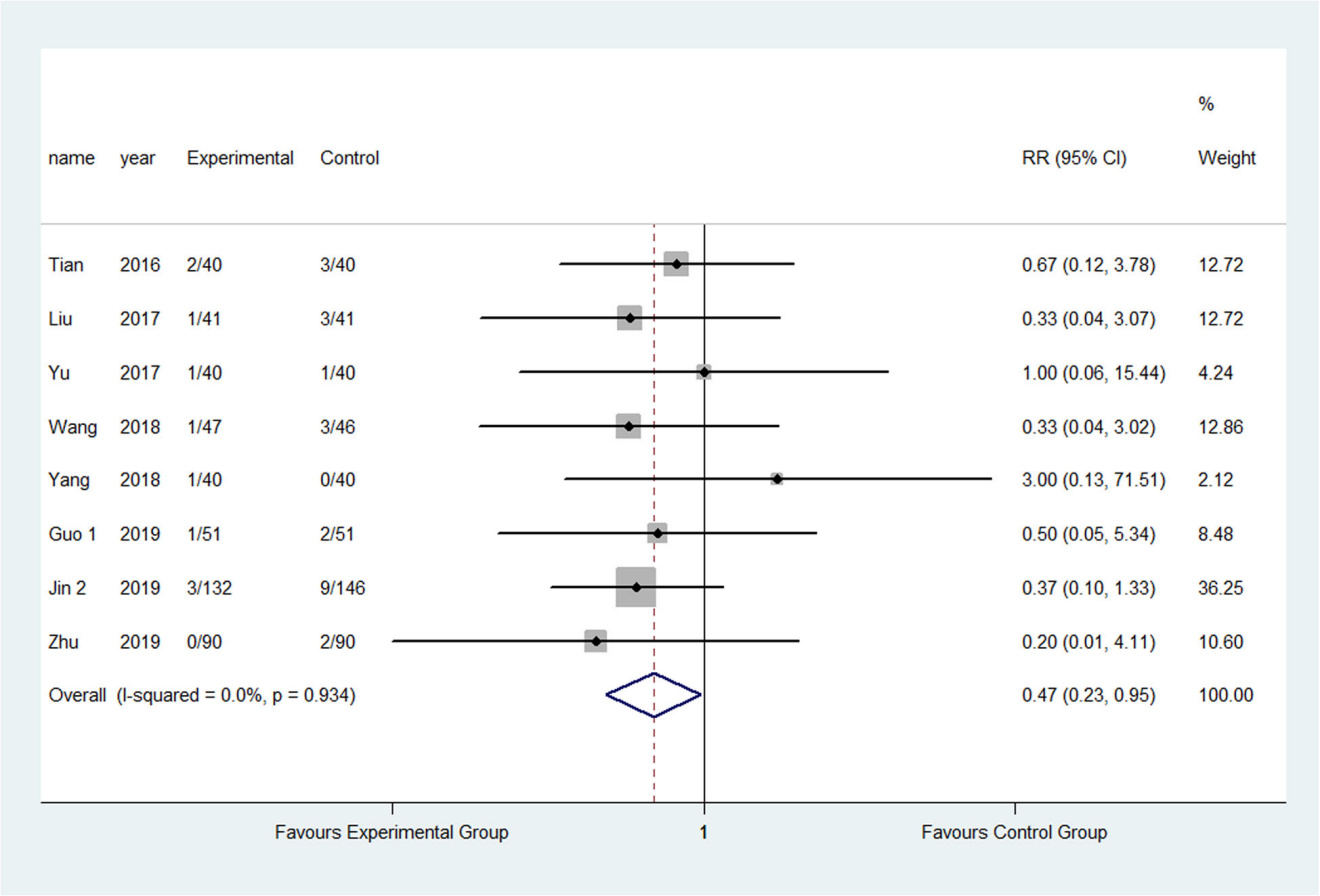
Incidence of incision infection between the experimental group and the control group. RR risk ratio

**Fig. 15:**
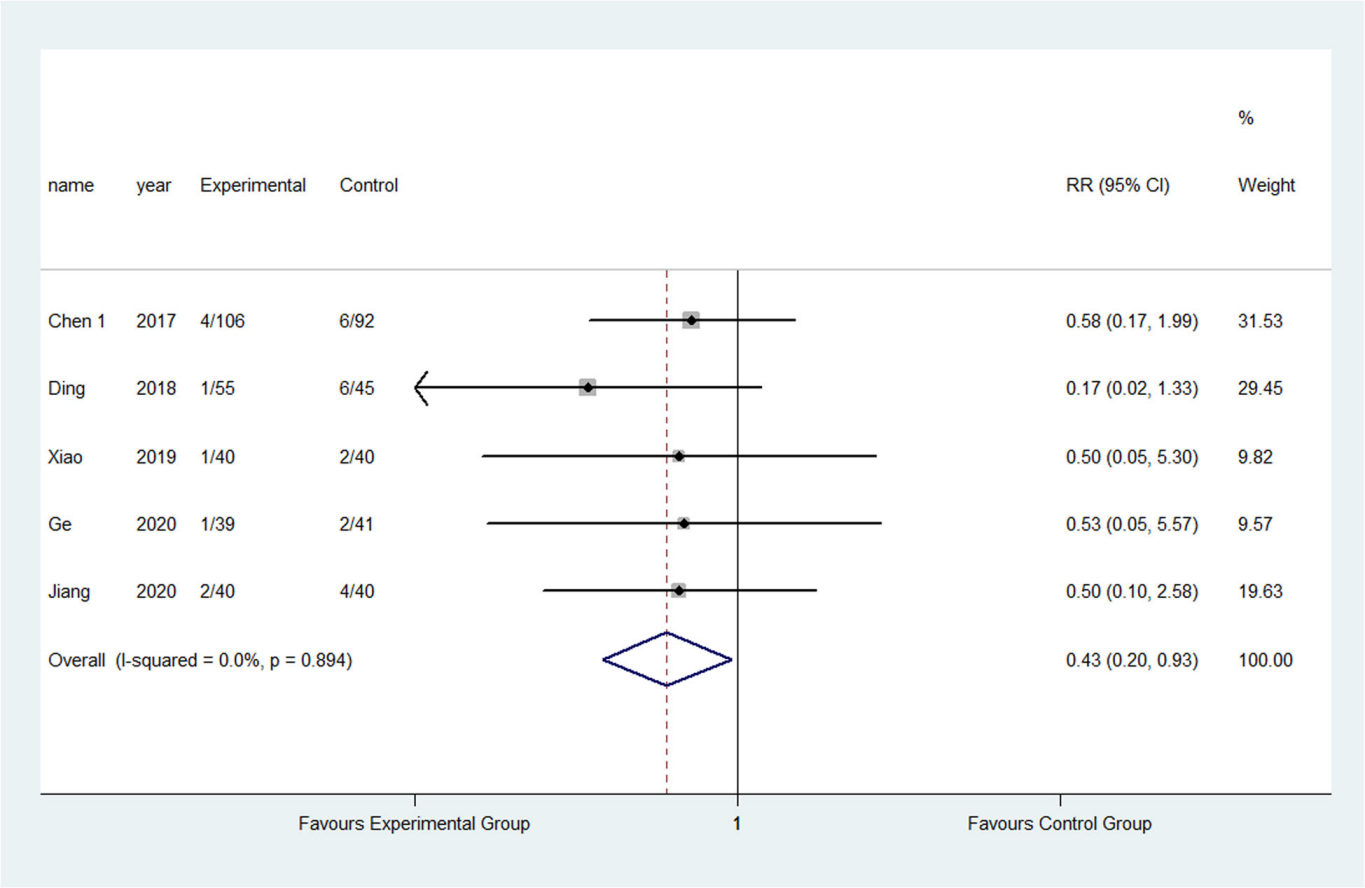
Incidence of constipation between the experimental group and the control group. RR risk ratio

**Fig. 16:**
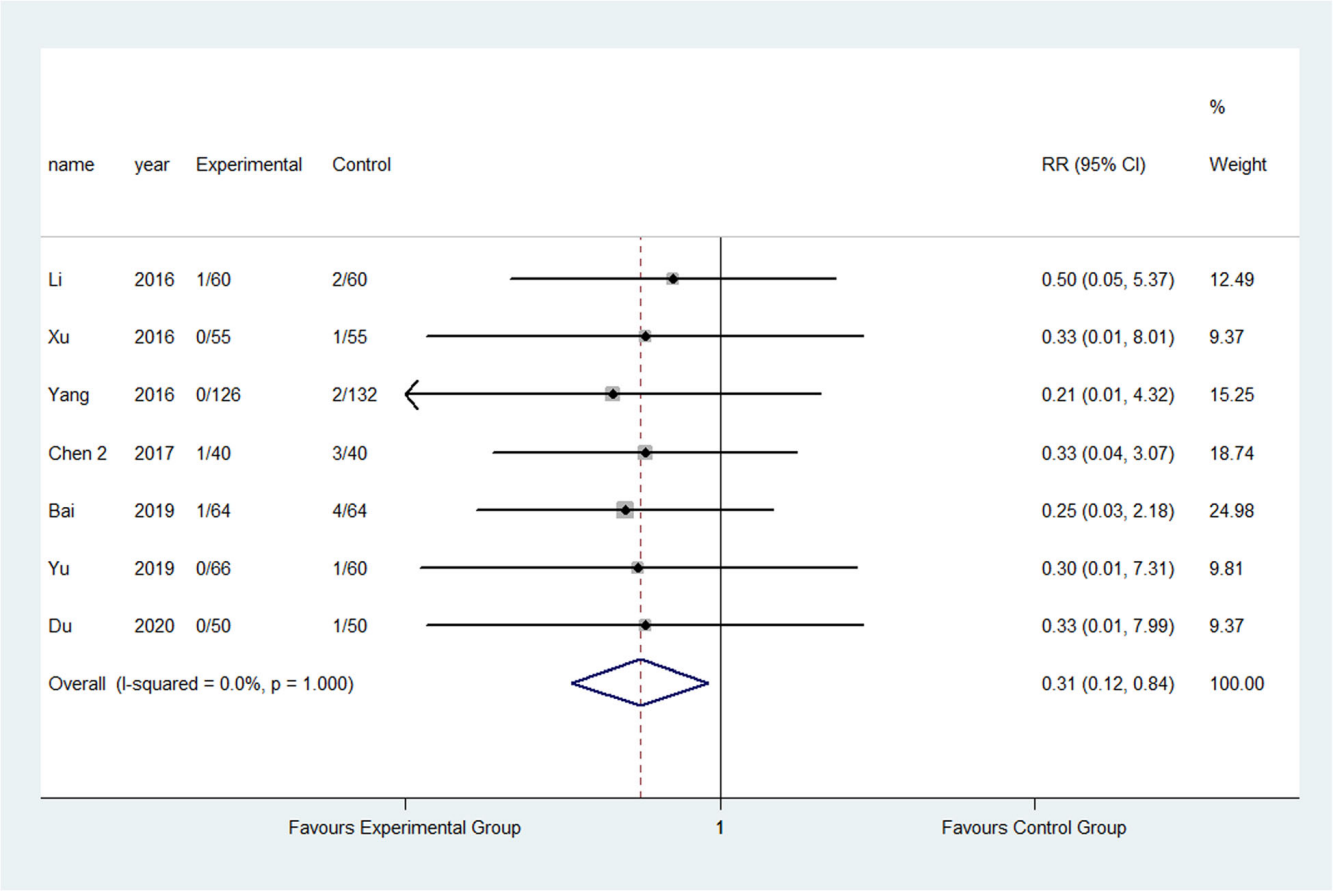
Incidence of dislocation of prosthesis between the experimental group and the control group. RR risk ratio

The result showed that compared to the control group, FTS could significantly reduce the incidence of respiratory system infection (3.52% vs 5.28%) (RR: 0.68, 95% CI 0.52 to 0.87, *I*^2^=20.7%); urinary system infection (2.22% vs 6.98%) (RR:0.33, 95% CI 0.21 to 0.52, *I*^2^=0.0%); VTE (1.13% vs 3.24%) (RR:0.40, 95% CI 0.29 to 0.56, *I*^2^=0.0%) ; pressure sore (2.50% vs 4.02%) (RR:0.63, 95% CI 0.47 to 0.86, *I*^2^=13.5%) ; incision infection (2.08% vs 4.66%) (RR:0.47, 95% CI 0.23 to 0.95, *I*^2^=0.0%); constipation (3.33% vs 7.75%) (RR:0.43, 95% CI 0.20 to 0.93, *I*^2^=0.0%) and prosthesis dislocation (0.65% vs 3.04%) (RR:0.31, 95% CI 0.12 to 0.84, *I*^2^=0.0%) as shown in Figs. 10, 11, 12, 13, 14, 15 and 16.

#### Publication bias and sensitivity analysis

The funnel plot showed that there was bias among retrieved articles as shown in Supply Fig. 1, 2, 3, 4, 5, 6, 7, 8, 9, 10, 11, 12 and 13.

## Discussion

Fast track surgery (FTS), also named as enhanced recovery after surgery (ERAS), improved or reselected partially perioperative steps of traditional hip arthroplasty in order to reduce the direct surgical injury, surgical stress response, and operation-related complication and achieve the purpose of promoting the rapid rehabilitation of patients, which could shorten the length of stay and reduce the cost of hospitalization [78–80]. The ultimate objective of FTS is to achieve “painless and risk-free surgery,” which has attracted worldwide attention [81].

Kehlet published an article to describe “fast-track surgery” (also known as enhanced recovery after surgery, ERAS) in 2001 first. The ERAS society, a non-profit and multidisciplinary medical association, was founded later that same year, aimed to help worldwide to carry through ERAS Social Guidelines systematically, and it has remained a challenge to monitor patient compliance and follow-up the rehabilitation outcomes [82]. The UK’s National Hip Fracture Database (NHFD), which collected information about fractured neck of femur, was established in 2007 and the most recent report supported early rehabilitation exercise [83]. NHFD has been focus on orthogeriatric assessment recently, an assessment before operation similar to the ERAS principles [84].

Nowadays, preoperative management of diabetes and anemia, personalized risk assessment, path sharing between enhanced recovery and hospitals, individualized pain management, multimodal anesthesia strategies, and the great differences in nursing in different area and so on still have room for improvement, which limited the promotion of FTS in worldwide [85]. Meanwhile, the research on ERAS has gradually shifted from standard indicators such as mortality, morbidity, and readmission rate to patient-reported outcome measures (PROMs).

The results we got consist with Husted et al.’s [86] (a prospective study), Larsson et al.’s [13] (a retrospective pilot study), and Bao et al.’s ( a meta-analysis), which suggested that patients could leave bed, recover the function of hip joint faster, suffer less pain, and reduce the incidence of postoperative complications, which could achieve the purpose of leaving hospital as soon as possible in addition after FTS. The novel nursing care delivery system also has a better satisfaction. However, the focus of their RCTs was to shorten the preparation time and nursing time, which means the results they come to may be influenced by shorter waiting times for surgery and cannot be attributed solely to the improvement of the chains of nursing. Bao et al. included a portion of studies with a lower quality, which might be measurement bias and selective bias, besides, the meta-analysis only analyzed the perioperative pain management of FTS [87]. Pollmann et al. [88] suggested that although the introduction of fast-track care for hip fractures significantly reduced length of stay, duration of surgery and risk of reoperation within 30 days, there was no significant different in 30-day, 90-day, or 1-year mortality rates. The data of the study were obtained retrospectively from electronic hospital records so that we could not identified that whether the patients follow the nursing protocol strictly. About half of the data on the admission are unclear, the others were admitted through fast-track admission pathway, which may have an influence on the outcome of mortality. The study by Amlie et al. reported that compared with the standard THA patients, patients with FTS regimen had an increased risk of postoperative revision surgery due to deep infection [89]. Therefore, there is still a controversial about whether FTS is effective and safe enough in the perioperative period for elderly patients with hip fracture.

At the time of assessment of the primary end point, a subgroup analysis was performed based on follow-up time. Subgroup analysis is performed only if each subgroup has more than two experimental comparisons. The result of follow-up time subgroup showed that patients’ Harris hip joint function score 6 months after surgery in the experimental group was significantly higher than that in control group, though the not applicable group is the highest. It could be concluded that FTS improves the long-term functional recovery of patients after hip replacement. The VAS in experimental group was significantly less than that in the control group 1 month after surgery.

As the primary endpoint, we evaluated was highly heterogeneous, and we did sensitivity analyses to decompose it. The results showed that after excluding Jia et al.’s article [59], and the overall effect of LOS has been affected significantly. We speculated that that the control group in research also offer a guidance of fracture functional exercise postoperative. There is also a great deal of influence of the overall effect of respiratory, VTE, and pressure sores after excluding Borges et al.’s article [72]. However, the RCT focused on accelerated surgical treatment (within 6 h), so that the waiting time for surgery is shorter than that of other patients, which may lead to heterogeneity.

The potential clinical implications of this meta-analysis are as follows: (1) 57 RCTs were retrieved which included a large sample size of 8886 participants compared to previous studies. (2) Subgroup analyses were performed according to the follow-up period to explain the influence of different factors on the overall effect. (3) We evaluated 12 indicators, including length of stay (LOS), Harris hip joint function score, VAS, satisfaction, the leaving bed time, respiratory system infection, urinary system infection, VTE, pressure sore, incision infection, constipation, and prosthesis dislocation, which were more comprehensive than previous articles.

The limitations of this study are as follows: (1) Most of the elderly patients with hip fractures were associated with risk factors (such as elder age, smoking), other complications (such as diabetes, hypertension), or some drug use history, which were adverse to incision healing. The influence of these baseline factors was not excluded and may lead to mixed bias. (2) We use the outcomes from reported events retrieved to integrate the results of this meta-analysis, so it is difficult to assess the impact of these baseline characteristics on the result. (3) Due to the limitations of the included study, this study was unable to explore the interaction between subgroup analyses. (4) The detail of the intervention measures in the control group (or standard track group) was not acceptable in most articles. (5) Only 3 of the retrieved articles were published in English journals and 54 in Chinese journals. Therefore, we have correctly attempt to address this problem by assessing the quality of the studies retrieved and rating most of them B.

This meta-analysis reveals that FTS could significantly shorten the LOS and reduce VAS, the leaving bed time, and the hospitalization costs and improve hip function. The incidence of complications (such as respiratory system infection, urinary system infection, VTE, pressure sore, incision infection, constipation, and prosthesis dislocation) also has been decreased significantly. Meanwhile, FTS improved patients’ satisfaction apparently. Its efficacy and safety were proved to be reliable.

Due to individual differences of every patient, the fast track should be adjusted clinically so that patients with special needs or high co-morbidity burden should be transferred to a safe and effective FTS [84]. A people-oriented approach is an important factor in optimizing care from surgical decisions to rehabilitation [85].

In the future, the perioperative strategy of rapid rehabilitation surgery in total hip replacement will need to extract more large samples of high quality for evidence-based analysis under a more perfect unified standard, so as to conduct more safe and effective multidisciplinary cooperation.

## Supplementary Information

**Additional file 1: Supply Figure 1.** Comparison of LOS between the experimental group and the control group. (funnel plot). SMD= standardized mean difference.

**Additional file 2: Supply Figure 2.** Comparison of Harris hip joint function score between the experimental group and the control group. (funnel plot). SMD= standardized mean difference.

**Additional file 3: Supply Figure 3.** Comparison of VAS between the experimental group and the control group. (funnel plot). SMD= standardized mean difference.

**Additional file 4: Supply Figure 4.** Comparison of satisfaction between the experimental group and the control group. (funnel plot). RR= Risk Ratio.

**Additional file 5: Supply Figure 5.** Comparison of the leaving bed time between the experimental group and the control group. (funnel plot). SMD= standardized mean difference.

**Additional file 6: Supply Figure 6.** Comparison of cost between the experimental group and the control group. (funnel plot). SMD= standardized mean difference.

**Additional file 7: Supply Figure 7.** Incidence of respiratory infection between the experimental group and the control group. (funnel plot). RR= Risk Ratio.

**Additional file 8: Supply Figure 8.** Incidence of urinary tract infection between the experimental group and the control group. (funnel plot). RR= Risk Ratio.

**Additional file 9: Supply Figure 9.** Incidence of VTE between the experimental group and the control group. (funnel plot). RR= Risk Ratio; VTE= venous thrombus embolism.

**Additional file 10: Supply Figure 10.** Incidence of pressure sores between the experimental group and the control group. (funnel plot). RR= Risk Ratio.

**Additional file 11: Supply Figure 11.** Incidence of incision infection between the experimental group and the control group. (funnel plot). RR= Risk Ratio.

**Additional file 12: Supply Figure 12.** Incidence of constipation between the experimental group and the control group. (funnel plot). RR= Risk Ratio.

**Additional file 13: Supply Figure 13.** Incidence of dislocation of prosthesis between the experimental group and the control group. (funnel plot). RR= Risk Ratio.

## Data Availability

None.

## Abbreviations

RCT: Randomized controlled trials
RR: Risk ratios
SMD: Standard mean difference
CI: Confidence intervals
HA: Hip arthroplasty
FTS: Fast track surgery
ERAS: Enhanced recovery after surgery
LOS: Length of stay
VAS: Visual analog scale

## References

[1] Greenlee WE (2006). Hip prosthesis and the use thereof.

[2] Bureau MN, Legoux JG, Denault J (2009). Implantable biomimetic prosthetic bone.

[3] Claire, Tilbury, Tsjitske (2016). Unfulfilled expectations after total hip and knee arthroplasty surgery: there is a need for better preoperative patient information and education. J Arthroplast.

[4] Gouvas N, Tan E, Windsor A (2009). Fast-track vs standard care in colorectal surgery: a meta-analysis update. Int J Color Dis.

[5] Jiang ZW, Li JT (2016). The present situation and prospect of accelerated rehabilitation surgery. (In Chinese) Chin J Surg.

[6] Kehlet H, Wilmore DW (2008). Evidence-based surgical care and the evolution of fast-track surgery. Ann Surg.

[7] Pissetti VC, Nunes RD, Zomer MT (2017). Fast-track surgery in intestinal deep infiltrative endometriosis. J Endometriosis Pelvic Pain Disorders.

[8] Lili W, Guichun J, Hui LI. The application effect of fast track surgery combined with psychological guidance on patients with radical resection of colon cancer. J Int Psychiatry. 2019.

[9] Yan S, Wenhui LI. Effect of fast track surgery concept on the postoperative recovery, psychological and physiological stress response in patients undergoing coronary artery bypass surgery. Chin J Health Psychol. 2019.

[10] Louise C, Burgess, Joe, et al. The effect of preoperative education on psychological, clinical and economic outcomes in elective spinal surgery: a systematic review. Healthcare (Basel, Switzerland). 2019;7. 10.3390/healthcare7010048 PMC6473918 30901875

[11] Kremer M, Ulrich A, Büchler MW (2005). Fast-track surgery: the Heidelberg experience.

[12] Kristensen MT, Kehlet H (2012). Most patients regain prefracture basic mobility after hip fracture surgery in a fast-track programme. Dan Med J.

[13] Larsson G, Holgers KM (2011). Fast-track care for patients with suspected hip fracture. Injury..

[14] Pan Y, Wang H, Wu B, et al. Application of the concept of fast track surgery in colorectal surgery. Anti-Tumor Pharmacy. 2011.

[15] Sharrock NE (2000). Fast-track anaesthesia and postoperative care: orthopaedic surgery.

[16] Husted H, Otte KS, Kristensen BB (2010). Readmissions after fast-track hip and knee arthroplasty. Arch Orthop Trauma Surg.

[17] Eriksson M, Kelly-Pettersson P, Stark A (2012). ‘Straight to bed’ for hip-fracture patients: a prospective observational cohort study of two fast-track systems in 415 hips. Injury-Int J Care Injured.

[18] Complications and patient-reported outcome after hip fracture (2015). A consecutive annual cohort study of 664 patients. Injury-Int J Care Injured.

[19] Haugan K, Johnsen LG, Basso T (2017). Mortality and readmission following hip fracture surgery: a retrospective study comparing conventional and fast-track care. BMJ Open.

[20] Vrabel M (2009). Preferred Reporting Items for Systematic Reviews and Meta-Analyses: the PRISMA statement. Rev Esp Nutr Hum Diet.

[21] Li Y, Yu Y, Xue H (2016). The present situation and Prospect of accelerated rehabilitation surgery. Med Innov China.

[22] Tian XZ, Zhang JH (2016). Observation on the effect of the concept of rapid rehabilitation surgery in hip surgery in the elderly. World Latest Med Inf.

[23] Xu HP, Zhao H, Liu YJ (2016). Clinical implementation of rapid rehabilitation program for elderly patients with hip fracture. J Nurs Sci.

[24] Yang G, Chen W, Chen W (2016). Feasibility and safety of two-day discharge after fast-track total hip arthroplasty: a Chinese experience. J Arthroplast.

[25] Zhang YZ, Shen CP (2016). Nursing analysis of 56 cases of senile hip fracture with the concept of rapid rehabilitation surgery. Today Nurse.

[26] Chen J, Zi JH. Application of ERAS nursing model in perioperative period of elderly patients with hip fracture. (In Chinese) J Qilu Nurs. 2017;(16):19–21.

[27] Chen M, Zheng YT, Tang Q (2017). Effect of accelerated rehabilitation surgical nursing on complications and function after hip arthroplasty. Chin Gen Pract Nurs.

[28] Fu HQ (2017). Application of the concept of accelerated rehabilitation surgery in perioperative nursing of senile hip fracture patients. Chin J New Clin Med.

[29] Li N, Huang P (2017). The practical application of the idea of rapid rehabilitation surgery in the nursing of senile patients with artificial hip replacement. Chin Baby.

[30] Liu YM (2017). Evaluation of the effect of rapid rehabilitation surgery on the quality of nursing care in hip joint surgery. (In Chinese) World Chin Med.

[31] Wan C (2017). Observation on the effect of rapid rehabilitation on nursing quality of hip joint. Yin Shi Bao Jiang.

[32] Wei R (2017). Application of rapid rehabilitation surgery in nursing care of patients with peri-hip fracture. J Nantong Univ (Med Sci).

[33] Yu JP, Zhao R, Sun Y. Observation on the effect of rapid rehabilitation surgery on nursing quality of hip joint surgery. Wield Chin Med. 2017;(a01):180–1.

[34] Zhang XH (2017). Application of the concept of rapid rehabilitation surgery in nursing care of hip arthroplasty. J Clinic Nurs Practicality.

[35] Zou XY, Fang X. Clinical application of nursing concept of rapid rehabilitation surgery in postoperative nursing of hip fracture in the elderly. For All Health. 2017.

[36] Ding YH (2018). Application of the concept of rapid rehabilitation surgery in nursing care of hip arthroplasty. Home Med.

[37] Jin Y, Tian J, Xie JL, Li XY (2018). Talking about the effect of rapid rehabilitation nursing on elderly patients with hip fracture. World Latest Med Inf.

[38] Li CB, Yao M (2018). Application of rapid rehabilitation nursing model in patients undergoing artificial hip arthroplasty. J Med Aesthetics Cosmetol.

[39] Li XY (2018). Application and effect analysis of accelerated rehabilitation surgery concept in perioperative nursing care of elderly patients with hip fracture. For All Health.

[40] Liu XT (2018). Application of accelerated rehabilitation surgery in perioperative nursing care of elderly patients with hip fracture. China Health Vison.

[41] Qian Z, Yang ZY, Wang B (2018). Discussion on rapid rehabilitation nursing of elderly patients with hip fracture. J Clin Med Pract.

[42] Wang XJ (2018). Observation on the effect of accelerated rehabilitation in perioperative nursing of hip arthroplasty. J Clinic Nurs Practicality.

[43] Yang HJ. Application of the concept of rapid rehabilitation surgery in nursing care of hip arthroplasty. J Hunan Univ Chin Med. 2018;(a01):745–6.

[44] Yang ZH, Zhu SX, Ye L, Wang MM, Wang SQ (2018). Application of accelerated rehabilitation surgery in artificial hip arthroplasty with lumbar plexus-lumbar paraspinal nerve block. Guangdong Med J.

[45] You HY, Xia X (2018). Application of rapid rehabilitation nursing model in elderly patients with hip fracture. Doctor..

[46] Zang QQ (2018). Observation on the effect of nursing model of enhanced recovery after surgery (ERAS) in perioperative period of elderly patients with hip fracture. China Health Care Nutr.

[47] Zhai YJ, Zhang AR, Wei HD, Gu YJ (2018). Evaluation of the value of accelerated rehabilitation surgery in perioperative nursing care of elderly patients with hip fracture. Yin Shi Bao Jiang.

[48] Zheng YJ (2018). Analysis of the value of rapid rehabilitation nursing in postoperative rehabilitation of patients undergoing hip arthroplasty. Yin Shi Bao Jiang.

[49] Zuo JJ (2018). Analysis of the value of rapid rehabilitation nursing in postoperative rehabilitation of patients undergoing hip arthroplasty. J Shandong Med Coll.

[50] Bai X (2019). Rapid rehabilitation nursing in perioperative period of hip arthroplasty. Yin Shi Bao Jiang.

[51] Bao T, Zhu JX (2019). Application of accelerated Rehabilitation surgery in Hip fracture of the elderly in Orthopaedics. Special Health.

[52] Chen JP (2019). Observation on the effect of the concept of rapid rehabilitation surgery in patients undergoing hip surgery. China Health Vision.

[53] Francesco, Fusco, Helen, et al. Rehabilitation after resurfacing hip arthroplasty: cost-utility analysis alongside a randomized controlled trial. Clin Rehabil 2019;33(6):1003-14. 10.1177/0269215519827628 30747010

[54] Guo KK, Liang X, Zhou L, He LQ (2019). Analysis of perioperative nursing effect of senile hip fracture with the concept of rapid rehabilitation surgery. Med Front.

[55] Guo N (2019). Analysis of perioperative nursing effect of senile hip fracture with the concept of rapid rehabilitation surgery. China Health Care Nutr.

[56] He W (2019). Effect of rapid rehabilitation nursing on elderly patients with hip fracture after operation. Med J Chin Peoples Health.

[57] Huang CY (2019). Nursing experience of rapid rehabilitation in 40 patients after hip arthroplasty. Health Required.

[58] Jia YY, Peng GL, Yang MH, Liu Z (2019). Nursing experience of rapid rehabilitation in 40 patients after hip arthroplasty. Chin J Med.

[59] Jiang QQ, Hu M (2019). Analysis of the application of rapid rehabilitation nursing in the nursing of elderly patients with hip fracture. Yin Shi Bao Jiang.

[60] Jin Y, Li XY, Kuang YX, Yang HB (2019). Observation on the effect of rapid rehabilitation nursing model on elderly patients with hip fracture. World Latest Med Inf (Electron Version).

[61] Jing ZP, Zhu YC, Wang ZY, Xie HF, Feng B, Liu FW (2019). Application of accelerated rehabilitation surgical nursing based on nutrition support in elderly patients with hip fracture. Chin J Modern Nurs.

[62] Li ZX (2019). Application of perioperative nursing based on the concept of accelerated rehabilitation surgery in hip arthroplasty. Chin J Trauma Diasability Med.

[63] Liang SX. To explore the effect of nursing care of elderly patients with hip fracture using the concept of enhanced recovery after surgery (ERAS). (In Chinese) Smart Healthc. 2019;(25).

[64] Liu XM (2019). Analysis of the effect of rapid rehabilitation nursing for elderly patients with hip fracture treated by operation. Contemp Med Symp.

[65] Sun YJ. Observation on nursing care of elderly patients with hip fracture using the concept of enhanced recovery after surgery (ERAS). (In Chinese) World Latest Med Inf. 2019;(25).

[66] Xiao X, Li JH (2019). To explore the effect of nursing care of elderly patients with hip fracture using the concept of enhanced recovery after surgery (ERAS). Home Med.

[67] Yang LF (2019). Effect of rapid rehabilitation nursing in perioperative period of elderly patients with hip fracture. Electron J Clin Med Lit.

[68] Yu LL, Wang LZ, Shao QY, Wang Q (2019). The application of rapid rehabilitation nursing in green channel treatment of elderly patients with hip fracture. Tianjin J Nurs.

[69] Zhang YM (2019). Application of rapid rehabilitation concept in nursing care of elderly patients after artificial hip arthroplasty. Electron J Pract Clin Nurs Sci.

[70] Zhu L (2019). Study on the application of rapid rehabilitation surgical nursing model in the perioperative period of hip fracture in the elderly. Electron J Pract Clin Nurs Sci.

[71] Others THAI, Leung F, Fang C, et al. Accelerated surgery versus standard care in hip fracture (HIP ATTACK): an international, randomised, controlled trial. 2020. 10.1016/S0140-6736(20)30058-1 32050090

[72] Du L, Ma HF, Qiao JJ (2020). Application of accelerated rehabilitation surgery concept in perioperative nursing care of elderly patients undergoing hip arthroplasty. J Kunming Med Univ.

[73] Ge WW, Cai L, Yan XT. Observation on the effect of accelerated rehabilitation surgery in perioperative nursing of hip arthroplasty. Chin Remedies Clin. 2020;4.

[74] Jiang CY, Wang H, Huang WP, Li HZ (2020). Application of early out-of-bed intervention based on accelerated rehabilitation surgery in elderly patients with hip fracture. Chin J Mod.

[75] Liang MM, Guo L, Cong L (2020). Clinical application of accelerated rehabilitation surgery in perioperative nursing care of patients undergoing total hip arthroplasty. Chin J Pract Nurs.

[76] Yu FW (2020). Application of accelerated rehabilitation surgery in perioperative nursing of elderly patients with hip fracture. China Health Care Nutr.

[77] Zheng XF, Chen GX, Huang YR, Xia ZJ (2020). Effect of accelerated rehabilitation surgical nursing on complications and function after hip arthroplasty. Cap Med.

[78] Ehrlich, Kellokumpu, Wagner (2015). Comparison of laparoscopic and open colonic resection within fast-track and traditional perioperative care pathways: clinical outcomes and in-hospital costs. Scand J Surg.

[79] Shi W, Lisha LU, Shihui MA. Clinical curative observation of using Jiedu Zhixue decoction combined with functional exercise in promoting the rapid recovery of patients after total hip replacement. J Sichuan Tradit Chin Med. 2017.

[80] Gao C, Fu T, Liu X, et al. New advances of fast track surgery in pediatric surgery. Contemp Med. 2018.

[81] Wilmore DW, Kehlet H (2001). Management of patients in fast track surgery. Bmj..

[82] Johansen A, Boulton C, Hertz K, Ellis M, Burgon V, Rai S (2017). The National Hip Fracture Database (NHFD) – using a national clinical audit to raise standards of nursing care. Int J Orthop Trauma Nurs.

[83] Greenshields N, Mythen M. Enhanced recovery after surgery. Curr Anesthesiol Rep. 2020;10(1).

[84] Rapp SM (2010). Fast-track joint arthroplasty protocols increasing patient safety and satisfaction. Orthop Today Eur.

[85] Husted H, Holm G, Jacobsen S. Predictors of length of stay and patient satisfaction after hip and knee replacement surgery: fast-track experience in 712 patients. Acta Orthop. 2008. 10.1080/17453670710014941 18484241

[86] Bao XH, Hu YN, Zheng SX, Fu LQ, Yan SS (2019). A meta-analysis of fast-track surgery in perioperative pain managemen- of elderly patients with hip fracture. Nurs Integr Tradit Chin West Med.

[87] Berg U, Berg M, Rolfson O, et al. Fast-track program of elective joint replacement in hip and knee – Patients’ experiences of the clinical pathway and care process. J Orthop Surg Res. 2019;14(1). 10.1186/s13018-019-1232-8 PMC6587282 31227003

[88] Pollmann CT, Røtterud JH, Gjertsen JE (2019). Fast track hip fracture care and mortality – an observational study of 2230 patients. BMC Musculoskelet Disord.

[89] B?K HT (2017). Fast track in hip arthroplasty. Effort Open Rev.

