# Supplementary figures and images for: The efficacy and safety of fast track surgery (FTS) in patients after hip fracture surgery: a meta-analysis
Source: J Orthop Surg Res. 2021 Feb 27;16:162. doi: 10.1186/s13018-021-02277-w (PMC7913454; doi:10.1186/s13018-021-02277-w)

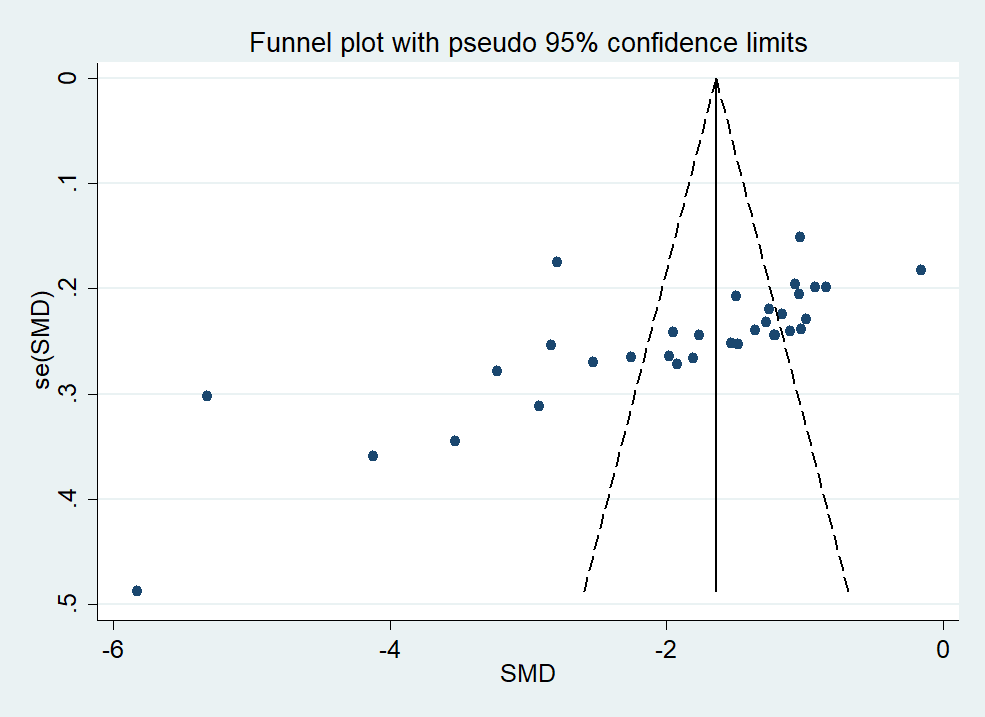

Supplement: Supplementary file 1 — Additional file 1: Supply Figure 1. Comparison of LOS between the experimental group and the control group. (funnel plot). SMD= standardized mean difference. [file 13018_2021_2277_MOESM1_ESM.tif]

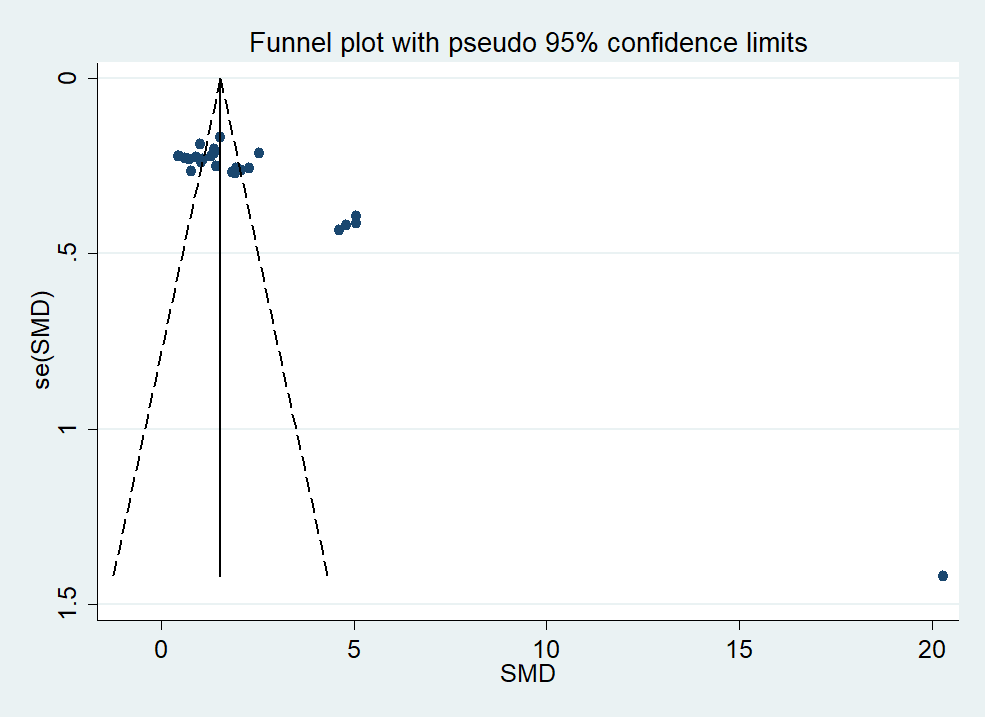

Supplement: Supplementary file 2 — Additional file 2: Supply Figure 2. Comparison of Harris hip joint function score between the experimental group and the control group. (funnel plot). SMD= standardized mean difference. [file 13018_2021_2277_MOESM2_ESM.tif]

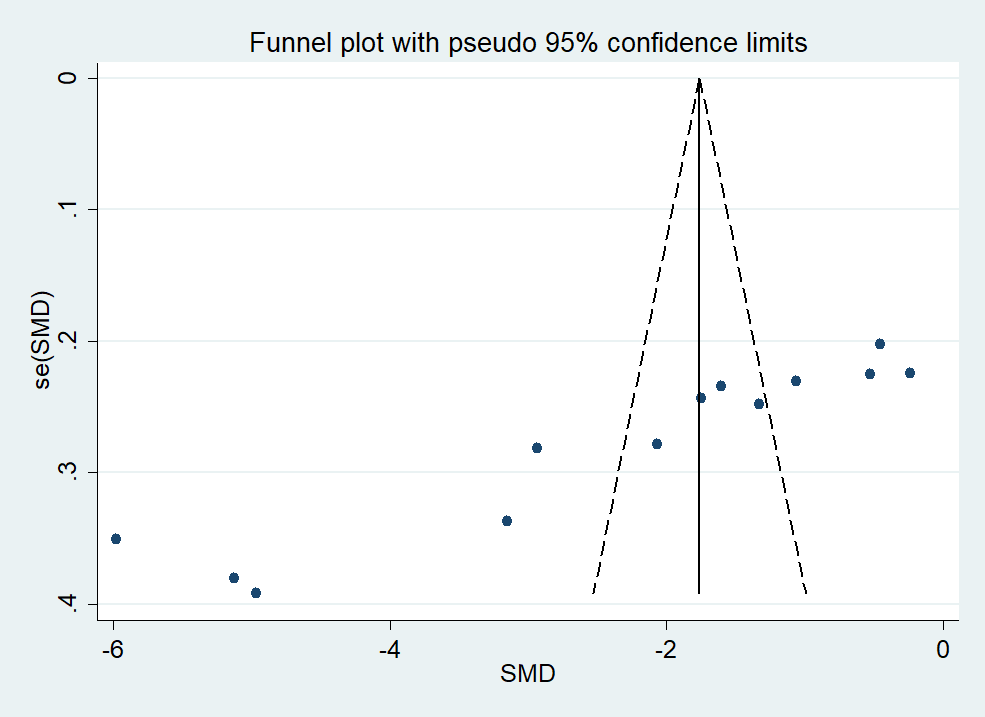

Supplement: Supplementary file 3 — Additional file 3: Supply Figure 3. Comparison of VAS between the experimental group and the control group. (funnel plot). SMD= standardized mean difference. [file 13018_2021_2277_MOESM3_ESM.tif]

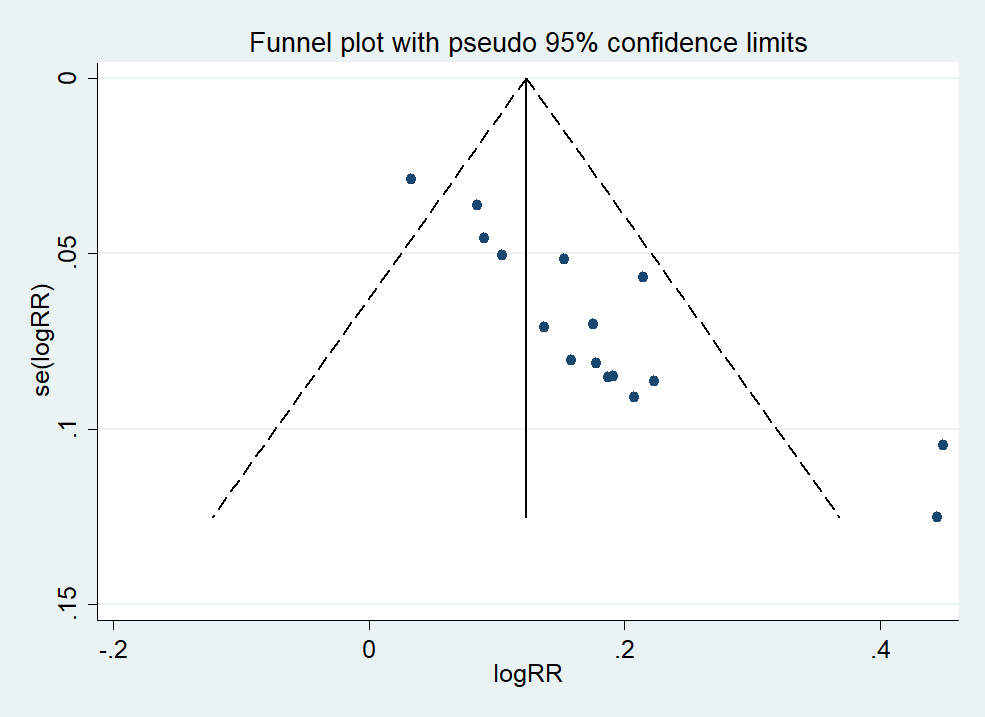

Supplement: Supplementary file 4 — Additional file 4: Supply Figure 4. Comparison of satisfaction between the experimental group and the control group. (funnel plot). RR= Risk Ratio. [file 13018_2021_2277_MOESM4_ESM.tif]

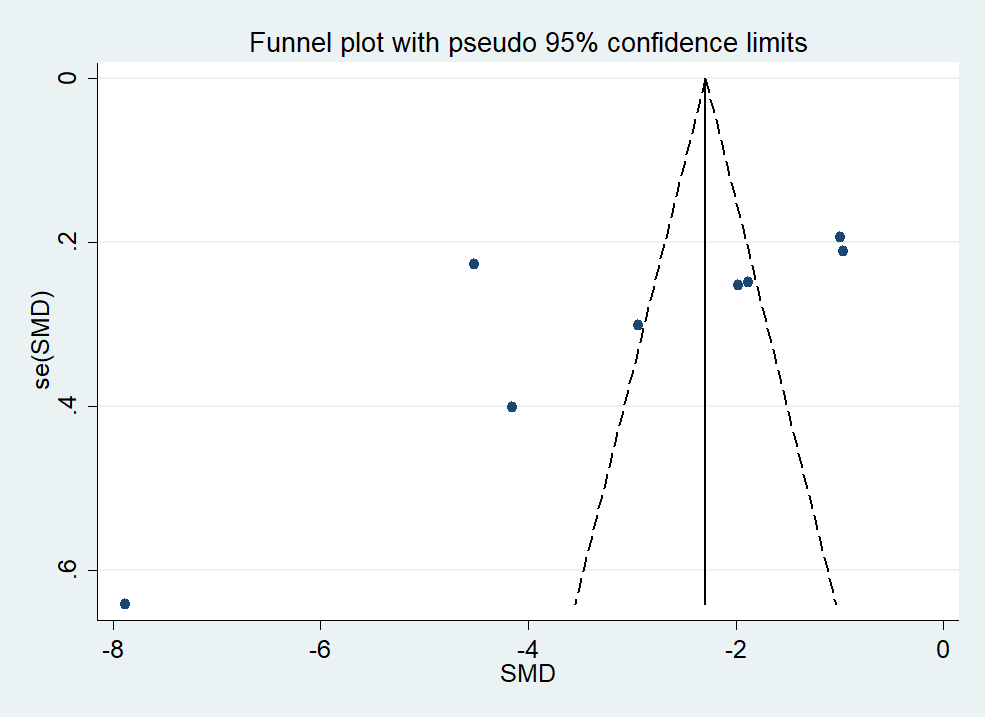

Supplement: Supplementary file 5 — Additional file 5: Supply Figure 5. Comparison of the leaving bed time between the experimental group and the control group. (funnel plot). SMD= standardized mean difference. [file 13018_2021_2277_MOESM5_ESM.tif]

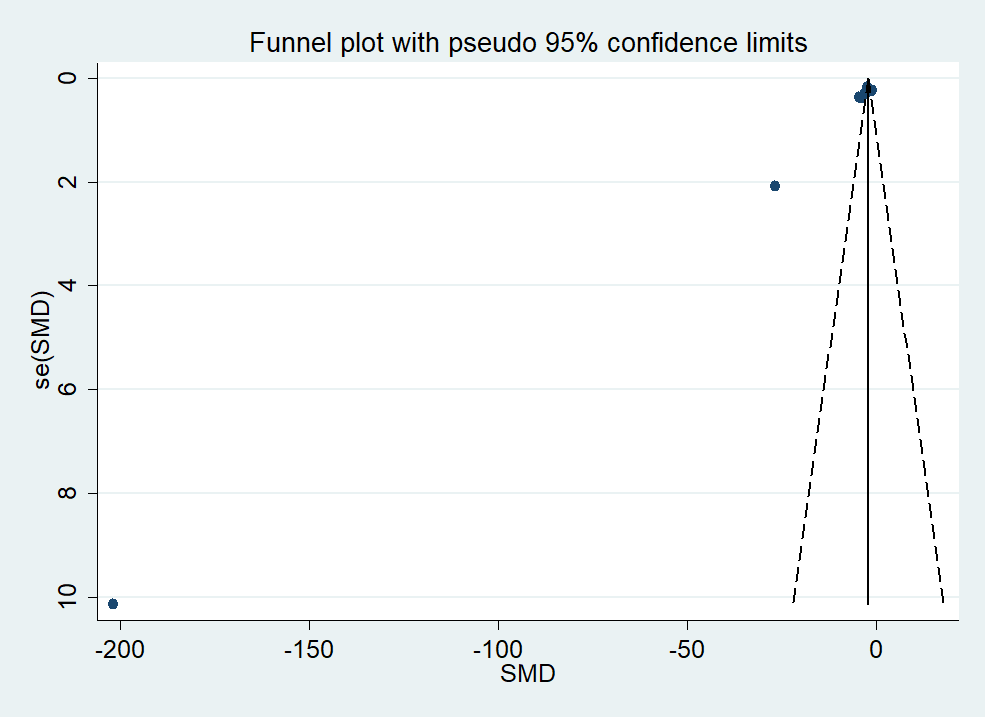

Supplement: Supplementary file 6 — Additional file 6: Supply Figure 6. Comparison of cost between the experimental group and the control group. (funnel plot). SMD= standardized mean difference. [file 13018_2021_2277_MOESM6_ESM.tif]

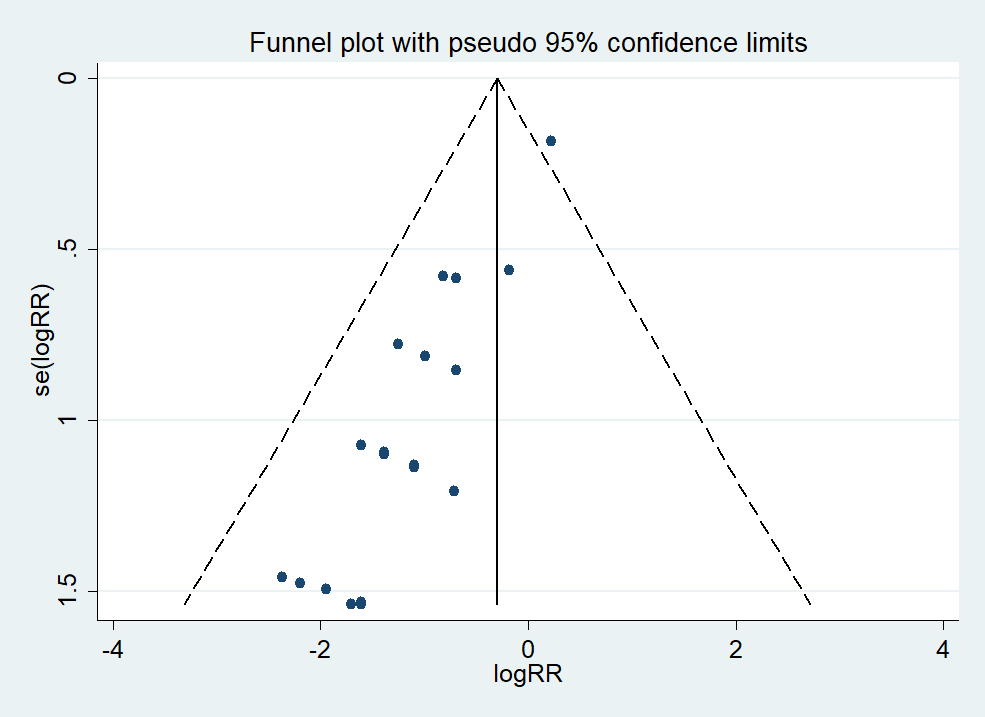

Supplement: Supplementary file 7 — Additional file 7: Supply Figure 7. Incidence of respiratory infection between the experimental group and the control group. (funnel plot). RR= Risk Ratio. [file 13018_2021_2277_MOESM7_ESM.tif]

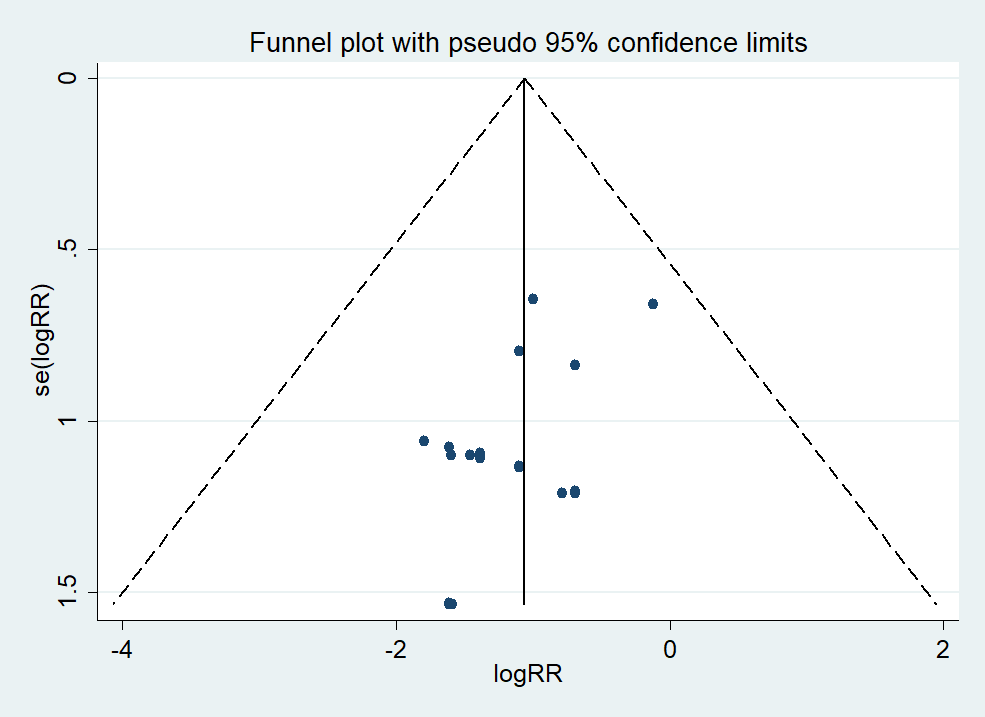

Supplement: Supplementary file 8 — Additional file 8: Supply Figure 8. Incidence of urinary tract infection between the experimental group and the control group. (funnel plot). RR= Risk Ratio. [file 13018_2021_2277_MOESM8_ESM.tif]

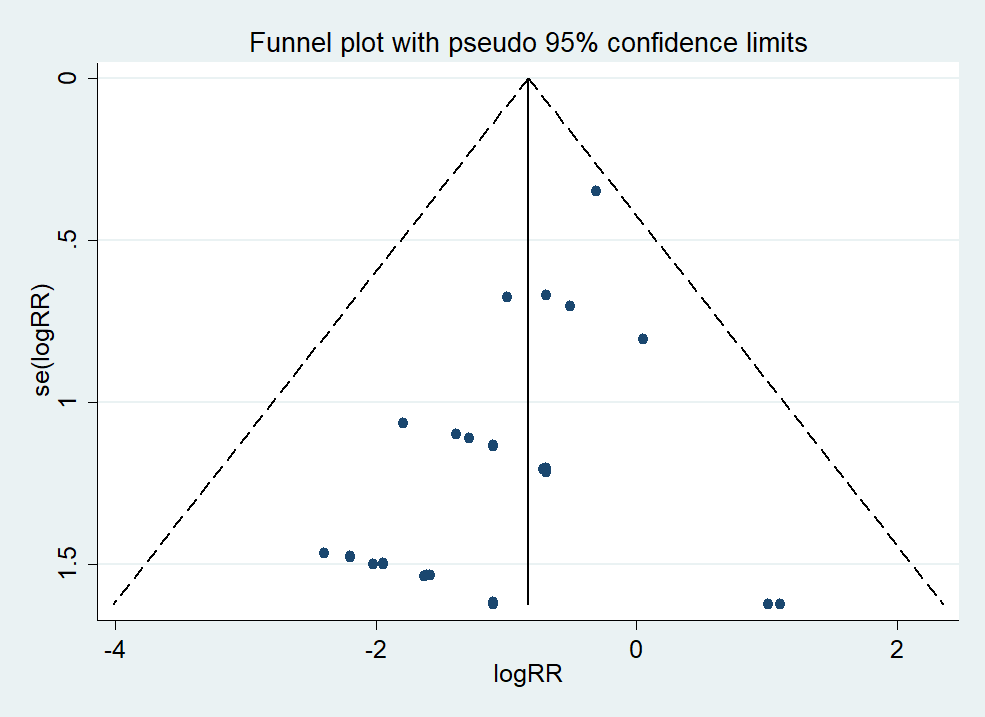

Supplement: Supplementary file 9 — Additional file 9: Supply Figure 9. Incidence of VTE between the experimental group and the control group. (funnel plot). RR= Risk Ratio; VTE= venous thrombus embolism. [file 13018_2021_2277_MOESM9_ESM.tif]

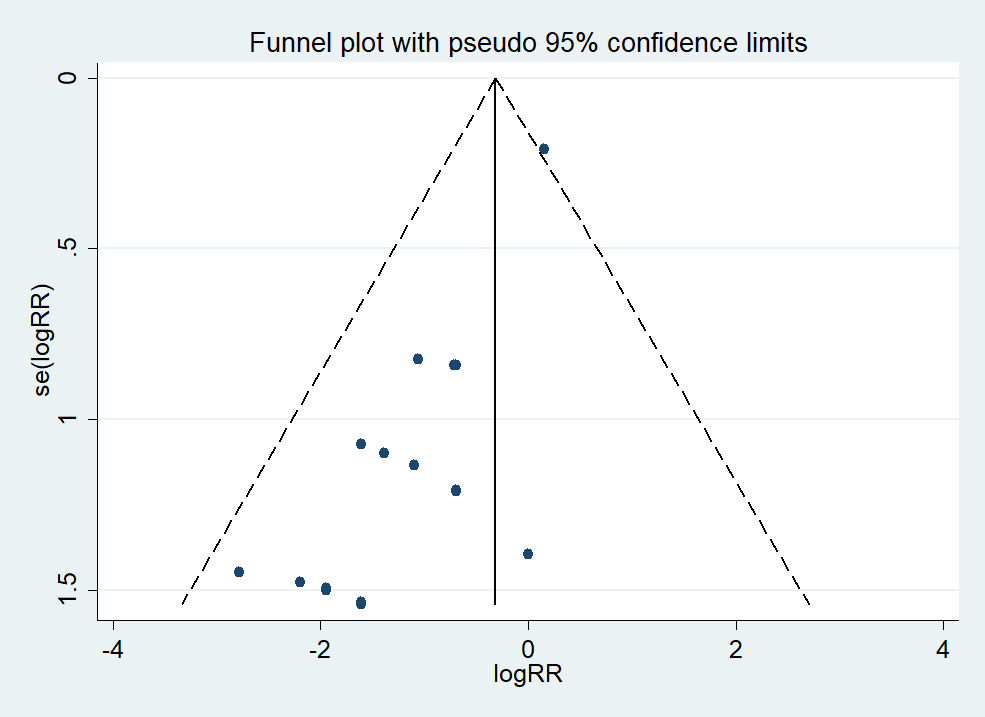

Supplement: Supplementary file 10 — Additional file 10: Supply Figure 10. Incidence of pressure sores between the experimental group and the control group. (funnel plot). RR= Risk Ratio. [file 13018_2021_2277_MOESM10_ESM.tif]

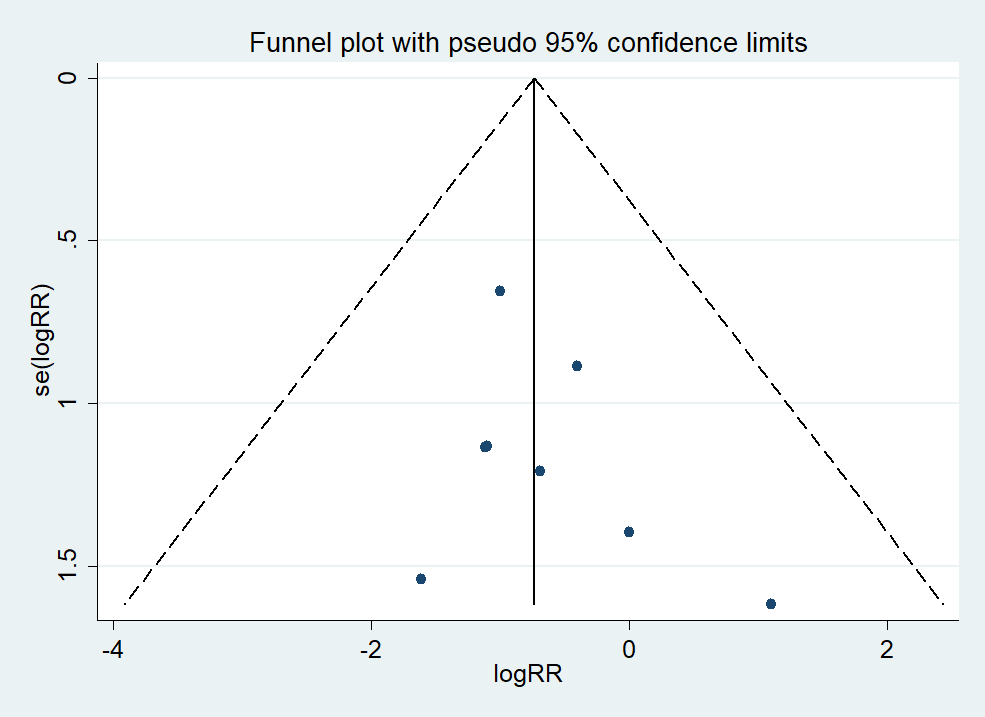

Supplement: Supplementary file 11 — Additional file 11: Supply Figure 11. Incidence of incision infection between the experimental group and the control group. (funnel plot). RR= Risk Ratio. [file 13018_2021_2277_MOESM11_ESM.tif]

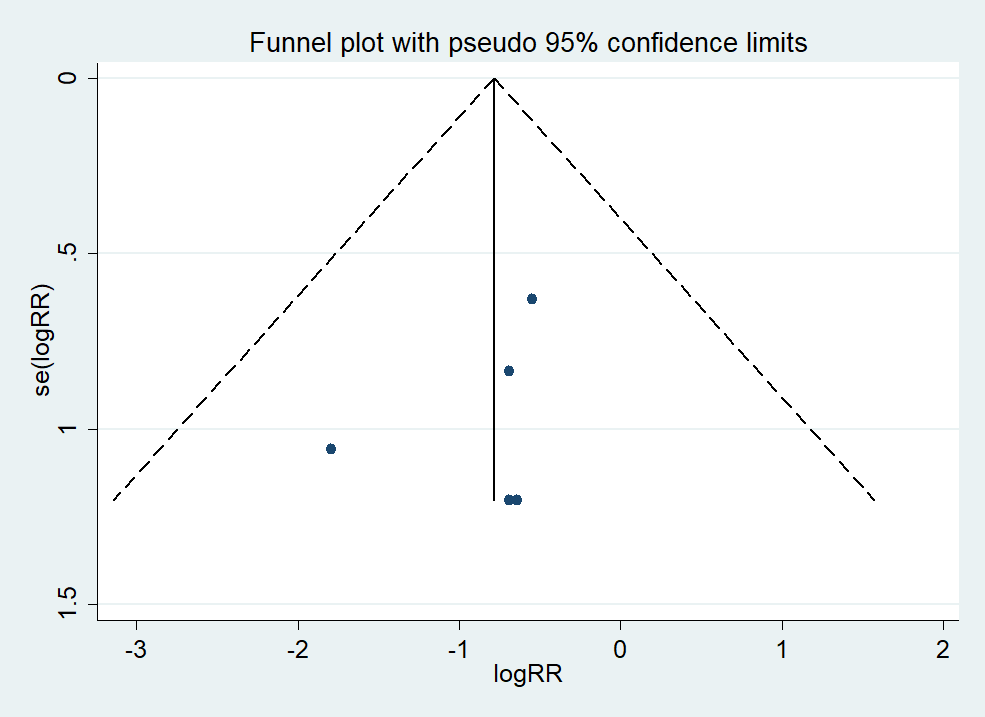

Supplement: Supplementary file 12 — Additional file 12: Supply Figure 12. Incidence of constipation between the experimental group and the control group. (funnel plot). RR= Risk Ratio. [file 13018_2021_2277_MOESM12_ESM.tif]

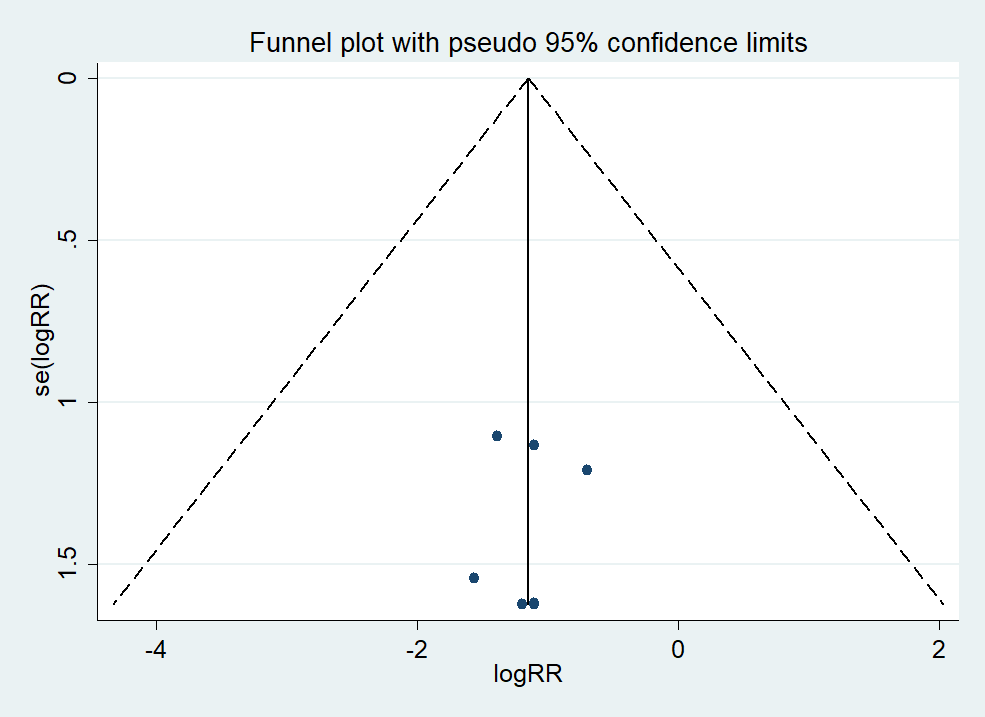

Supplement: Supplementary file 13 — Additional file 13: Supply Figure 13. Incidence of dislocation of prosthesis between the experimental group and the control group. (funnel plot). RR= Risk Ratio. [file 13018_2021_2277_MOESM13_ESM.tif]
